# Aptameric Probe Specifically Binding Protein Heterodimer Rather Than Monomers

**DOI:** 10.1002/advs.201900143

**Published:** 2019-04-09

**Authors:** Tao Bing, Luyao Shen, Junyan Wang, Linlin Wang, Xiangjun Liu, Nan Zhang, Xiao Xiao, Dihua Shangguan

**Affiliations:** ^1^ Beijing National Laboratory for Molecular Sciences Key Laboratory of Analytical Chemistry for Living Biosystems CAS Research/Education Center for Excellence in Molecular Sciences Institute of Chemistry Chinese Academy of Sciences Beijing 100190 China; ^2^ School of Chemical Sciences University of Chinese Academy of Sciences Beijing 100049 China

**Keywords:** alkaline phosphatase, aptamers, molecular probes, protein heterodimers, recognition

## Abstract

Dimerization of proteins occurs frequently and plays integral roles in biological processes. However, no single molecular probe is available for in situ detection of protein dimers on cells and tissues because of the difficulty of isolating complete protein dimers for probe preparation and screening, which has greatly hampered the biomedical study of protein dimers. Herein, a G‐rich DNA aptamer (termed BG2) that only binds alkaline phosphatase (AP) heterodimers rather than monomers is reported. This aptamer is generated by the cell‐SELEX (systematic evolution of ligands by exponential enrichment) technique and proves to fold into a duplex stabilized antiparallel G‐quadruplex structure. Using BG2 as molecular probe, AP heterodimers are found to be expressed on several kinds of cancer cells. As an affinity ligand, BG2 could isolate AP heterodimers from cell lysate. BG2 is also demonstrated to be applicable for tumor imaging in mice xenografted with cells highly expressing AP heterodimers. AP isozymes are found in several tissues and blood throughout the body, but the function and tissue distribution of AP heterodimers are totally unknown; therefore, BG2 could serve as a molecular probe to uncover the mystery of AP heterodimers. The generation of aptameric probes by cell‐SELEX will open up a new situation for the study of protein dimers.

## 1 Introduction

Dimerization of proteins occurs frequently in cells and plays important roles in various biological processes and cancer development.^1^ However, because most of the protein dimers are assembled by noncovalent protein–protein interactions, they are usually disassembled during cell lysis and protein isolation, making the detection and identification of protein dimers very difficult. Although some experimental techniques have been developed for isolation and identification of protein dimers, such as blue‐native polyacrylamide gel electrophoresis (PAGE), co‐immunoprecipitation, protein‐fragment complementation assays, mass spectrum, and X‐ray crystallography,^2^ the in situ detection of protein dimers on cells and tissues is still a serious challenge because of the lack of molecular probes for directly recognizing a protein dimer. For example, alkaline phosphatase (AP) homodimers and heterodimers formed by intestinal AP (IAP) and placental AP (PLAP) are reported several decades ago,^3^, ^4^, ^5^ but the distributions and functions of AP dimers on human cells and tissues are absolutely unknown due to lack of efficient probes for the direct recognition of AP dimers. Recently, great efforts have been made to visualize protein dimers on cells, resulting in the development of methods such as real‐time tracking receptor dimerization on live cells by single‐molecule fluorescence microscopy based on Förster resonance energy transfer between fluorescent protein‐fused receptors,^6^ imaging protein dimerization through proximity ligation, and proximity‐induced DNA assembly of two protein‐specific antibodies or aptamers.^7^ However, there is no method that could be used for in vivo or high throughput detection of protein dimers. To our knowledge, no single molecular probes (antibodies and aptamers) have been reported to recognize a protein dimer (heterodimer and homodimer) rather than its monomers.

**Scheme 1:**
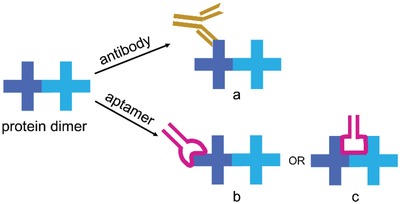
The possible binding models of protein dimers by antibodies and aptamers.

For single probe that can discriminate a protein dimer from its monomers, the probe should bind at a site formed by two monomers. Typically, antibodies are large Y‐shaped proteins (≈150 kDa), and they recognize a particular epitope on antigens with about 4–12 amino acids in size via the Fab's variable region (two tips of the “Y”) (**Scheme** **1**a).^8^ Therefore, antibodies are hard to bind an epitope spanning across a dimeric protein. In addition, antibodies are usually obtained by immunizing the animals with antigens. However, it is hard to obtain pure protein dimers to generate antibodies. Aptamers are synthetic single‐stranded oligonucleotides with high selectivity and specificity toward their targets.^9^ Compared with antibodies, aptamers are much smaller in size (≈6–30 kDa),^10^ which either bind a small epitope on target proteins^11^ (Scheme 1b) or fit in a binding pocket of large proteins or protein complexes. Therefore, aptamers may access to the pocket formed by a dimeric protein and interact with the adjacent amino acid residues of two monomers (Scheme 1c). Aptamers are generated by an iterative in vitro selection process known as systematic evolution of ligands by exponential enrichment (SELEX) against various targets, such as pure proteins, cells, and tissues.^9^ Selection using live cells as target (cell‐SELEX) is able to generate aptamers that recognize prior unknown molecular signatures on cell surface.^12^ Because proteins on live cells are present in their native and active states, cell‐SELEX has the potential to obtain aptamers that recognize unknown protein dimers or protein complexes.

Herein, we report an aptamer generated by cell‐SELEX technique that specifically bind alkaline phosphatase heterodimers rather than monomers. The applications of this aptamer for the detection and isolation of AP heterodimers were performed.

## 2 Results

### 2.1 Aptamer Generation and Characterization

**Figure 1:**
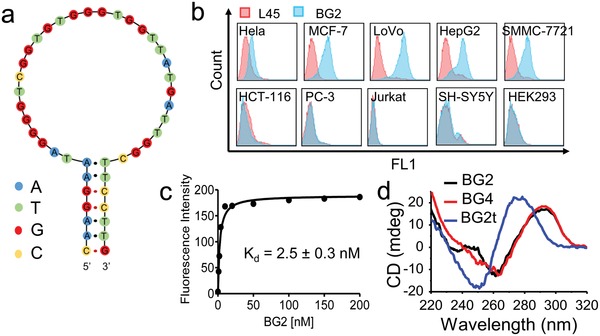
Characterization of aptamer BG2. a) Proposed secondary structure of aptamer BG2. b) The selectivity assay of BG2 to different cell lines. c) Binding curve of BG2 to LoVo cells determined by flow cytometry. d) CD spectra of BG2, BG4, and BG2t.

The aptamer was selected from a restricted library by using HeLa cells as target cells. The restricted library was favorable to form a duplex stabilized G‐quadruplex structure instead of a totally random library, which contained 5 G‐tracts in the central regions to favor the formation of G‐quadruplex, a YRRRRRR and a YYYYYYR sequence (Y = C or T; R = A or G) within the random region next to the primer binding regions to increase the probability of forming the duplex (see the Supporting Information for details). The cell‐SELEX was performed as previously described (see the Experimental Section and Figure S1 in the Supporting Information).^13^ After five rounds of selection, the enriched DNA pool of the fifth round was sequenced by high throughput sequencing. About 15 000 sequences were obtained, and 42% of them contained a conserved G‐rich motif, GGGGTCGGTGTGGGTGGTTATGATTGG, suggesting that it may be the binding motif of aptamers. The most abundant sequence, BG2 (**Figure** **1**a; Table S1, Supporting Information, not including the primer sequences), was chosen for further characterization.

The secondary structure prediction showed that BG2 adopted a hairpin structure with a G‐rich loop (Figure 1a). The binding assays showed that BG2 strongly bound to MCF‐7, HeLa, LoVo, HepG2, and SMMC‐7721 cell lines, whereas very weakly bound to HCT‐116, and nonbinding to PC‐3, Jurkat‐6E, SH‐SY5Y, and HEK293 cell lines (Figure 1b). Since BG2 showed the strongest binding to LoVo cell line, this cell line was mainly used for further study. The apparent equilibrium dissociation constant (*K*_d_) of BG2 was measured to be 2.5 ± 0.3 × 10^−9^ m (Figure 1c) by flow cytometry. BG2 could keep similar binding ability to LoVo cells at 4, 25, and 37 °C, even in the culture medium containing 10% FBS. In addition, BG2 also bound to fixed cells (Figure S2, Supporting Information).

In order to reveal the binding structure of BG2, a mutation assay was performed (Table S1, Supporting Information). Replacing the stem of BG2 with a new stem (BG4) caused its *K*_d_ increase to 11.5 ± 2.3 × 10^−9^ m (Figure S3a, Supporting Information). Furthermore, removing the stem of BG2 (BG2c) cause its *K*_d_ increase to 9.3 ± 1.6 × 10^−9^ m (Figure S3b, Supporting Information). Further removing the G‐tract at the 3′‐end and 5′‐end of BG2c respectively (BG2c2 and BG2c3) caused the loss of their binding ability to LoVo cells (Figure S3c, Supporting Information). Replacing the G‐tracts in the loop of BG2 with T‐tract (BG2t) or mutating G_16_ to A (BG2a) resulted in the loss of their binding ability. Deleting the TATGAT in the loop (BG2m) also caused the loss of its binding ability. These mutation assay results suggest that the G‐rich loop is the binding motif of BG2, the stem only stabilizes the binding structure, and the G‐tracts and the TATGAT sequences in the G‐rich loop are essential for aptamer binding.

To illuminate whether the G‐rich loop folds into G‐quadruplex, a circular dichroism (CD) spectral analysis was carried out (Figure 1d). The strong binding sequences, BG2 and BG4, showed a positive band around 295 nm and a negative band at 260 nm, indicating the formation of an antiparallel G‐quadruplex. The non‐G‐tract sequence, BG2t, did not show any CD signals of G‐quadruplex. The nonstem sequences, BG2c, BG2c2, and BG2c3, did not show clear CD signals of G‐quadruplex (Figure S3d, Supporting Information), suggesting that these sequences could not form stable and unique G‐quadruplexes without the help of stem. BG2m also showed the CD signals of antiparallel G‐quadruplex, but it did not bind to LoVo cells, suggesting that the removed sequence, TATGAT, may involve in the binding to target cells.

### 2.2 Target Identification of Aptamer BG2

The target protein of BG2 was identified by stable isotope labeling with amino acids in cell culture (SILAC)‐based quantitative proteomic analysis according to the protocol previously reported.^14^ After crosslinking of biotin‐labeled BG2 or control sequence BC1 to LoVo cells by 1% formaldehyde, the candidate proteins were captured from cell lysate by streptavidin‐coated sepharose beads, then digested with trypsin, and identified by LC‐MS/MS analysis. Totally 114 valid proteins were identified (Table S2, Supporting Information). According to the abundance ratio of proteins captured by aptamer BG2 and control sequence BC1, five membrane proteins with the ratio higher than 20 were speculated as the candidate protein targets, i.e., intestinal‐type AP, placental‐type AP, germ cell AP (GCAP), neural cell adhesion molecule L1 (L1CAM), and HLA class I histocompatibility antigen (HLA‐A). The MS quantification results for representative peptides of these proteins are shown in Figures S4–S8 in the Supporting Information.

Since three APs were identified with high SILAC ratio, and IAP and PLAP showed the highest score and sequence coverage in the candidate proteins, APs, especially IAP and PLAP, are the most possible targets of BG2. Furthermore, according to the information in ProteomicsDB (https://www.proteomicsdb.org), HLA‐A expressed in Jurkat and PC‐3 cell lines was higher than in HepG2 and MCF‐7 cell lines;^15^ however, our results showed that BG2 bound HepG2 and MCF‐7 cells but did not bind Jurkat and PC‐3 cells, suggesting that HLA‐A may not be the direct target of BG2. L1CAM was found highly expressed on surface of SH‐SY5Y cell line (Figure S9a, Supporting Information), but BG2 could not bind SH‐SY5Y at all (Figure 1b). When knocked down the expression of L1CAM on LoVo cells using small interfering RNA (siRNA), the BG2 binding was not affected (Figure S9b, Supporting Information), suggesting that L1CAM is not the direct target of BG2 either. Several reports have documented that IAP and PLAP can bind immunoglobulin;^16^ both L1CAM and HLA‐A are immunoglobulin‐like proteins, thus they might be isolated together with IAP and PLAP by BG2.

### 2.3 Discovery of AP Heterodimers as Molecular Target of BG2

**Figure 2:**
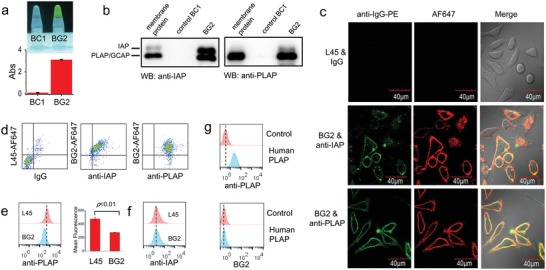
The binding of BG2 to APs. a) The AP activity of BG2 pull‐down proteins. b) Western blot analysis of proteins pulled down by control sequence (BC1) and BG2, respectively. c) Fluorescence imaging of live LoVo cells stained by BG2‐AF647 and anti‐IAP or anti‐PLAP. d) Flow cytometry assay of LoVo cells stained with BG2‐AF647 and anti‐IAP or anti‐PLAP. e) Competitive binding of anti‐PLAP (10 µg mL^−1^) toward LoVo cells by L45 (control) or BG2 (1 × 10^−6^ m). Data are presented as mean ± SD, unpaired two‐tailed *t*‐test, for BG2 versus L45 (*N* = 3). f) Competitive binding of anti‐IAP (10 µg mL^−1^) toward LoVo cells by L45 or BG2 (1 × 10^−6^ m). g) Flow cytometry assay of Jurkat cells anchored with human PLAP protein stained by anti‐PLAP or BG2‐FITC (top: control, down: anchored with human PLAP).

To confirm that APs are the target of BG2, we performed an aptamer pull‐down experiment and tested the pull‐down proteins using Alkaline Phosphatase Assay Kit. The BG2 pull‐down proteins showed very high AP activity (**Figure** **2**a), confirming that the molecular target is at least one isozyme of APs.

In order to figure out which AP is the real target of BG2, western blot assay and immunoassay by flow cytometry and confocal imaging were performed. It should be noticed that in the western blot assay, the commercially available anti‐IAP monoclonal antibodies (see the Supporting Information) showed strong cross‐reaction to PLAP because of the 85% homology between IAP and PLAP.^17^ The western blot assay showed that IAP and PLAP could be pulled down by aptamer BG2 but not by control sequences, BC1 (Figure 2b; Table S1, Supporting Information). The flow cytometry assay showed that both IAP and PLAP highly expressed on BG2‐binding cells (LoVo, HeLa, and MCF‐7), but lowly expressed on BG2‐nonbinding cells (Jurkat and PC3) (Figure S10, Supporting Information). The confocal imaging and flow cytometry showed that LoVo cells could be costained by BG2 (labeled with Alexa Fluor 647) and anti‐IAP/anti‐PLAP antibodies, respectively (Figure 2c,d). These results suggest that BG2 might bind both APs, i.e., IAP and PLAP, because of their high homology.

However, we noticed some differences between the costaining results of BG2/anti‐IAP and BG2/anti‐PLAP. In dot plots of flow cytometry, the fluorescence intensities of BG2 and anti‐IAP on cells showed a near linear relationship (Pearson's correlation coefficient = 0.703, *p* < 0.01), which suggests that BG2 and anti‐IAP might bind to different epitopes of an IAP (Figure 2d).^14^ But the costaining of BG2 and anti‐PLAP did not show the near linear relationship (Pearson's correlation coefficient = −0.016, *p* > 0.05). The competition experiment showed that the binding of anti‐PLAP to LoVo cells was partly diminished by the addition of excess amount of unlabeled BG2 (Figure 2e). To figure out whether BG2 binds to PLAP, we conducted competition experiment using purified human PLAP (P3895, Sigma). PLAP did not compete the binding of BG2 to LoVo cells at all (Figure S11, Supporting Information). In order to mimic the real environment of PLAP on cell surface, PLAP protein was anchored on the membrane of Jurkat cells (BG2 nonbinding cells). As shown in Figure 2f, anti‐PLAP bound the PLAP anchored Jurkat cells, but BG2 could not. These results suggest that PLAP may not be the molecular target of BG2, which contradict the above results.

**Figure 3:**
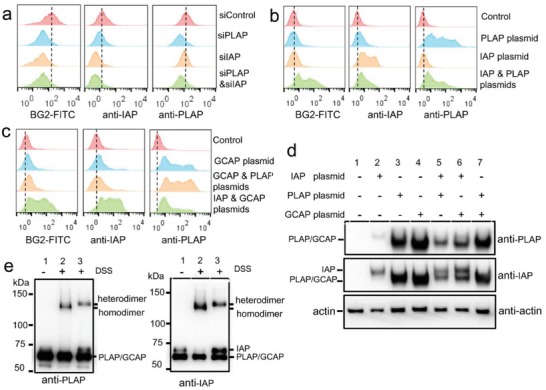
Identification of AP heterodimers as molecular target of BG2. a) The binding of BG2, anti‐IAP, and anti‐PLAP to AP knock down LoVo cells, binding toward LoVo cells was assessed at 72 h after siRNA treatment. b,c) The binding of BG2, anti‐IAP, and anti‐PLAP to PLAP, GCAP, or IAP overexpressed PC‐3 cells. d) Western blot assay of recombinant PLAP, GCAP, or IAP overexpressed on PC‐3 cells. PC‐3 cells were transfected with control plasmid (lane 1), IAP plasmid (lane 2), PLAP plasmid (lane 3), GCAP plasmid (lane 4), IAP + PLAP plasmids (lane 5), IAP + GCAP plasmids (lane 6), and PLAP + GCAP plasmids (lane 7). e) Western blot assay of AP dimers after in situ chemical crosslinking: lane 1, lysate of LoVo cells (uncrosslinked); lane 2, lysate of DSS crosslinked LoVo cells; lane 3, proteins pulled down by aptamer BG2 from lysate of DSS crosslinked LoVo cells.

In order to figure out the real target of BG2, we knocked down the expression of endogenous IAP and PLAP on LoVo cells, respectively, using siRNAs (**Figure** **3**a). Treatment of LoVo cells with siRNAs against IAP and PLAP, respectively, only reduced the binding of their corresponding antibody, and did not alter the binding of another antibody. However, treatment with siRNA against IAP, PLAP, or both of them significantly reduced the binding ability of BG2 to LoVo cells when compared with the treatment with control siRNAs. These results suggest that BG2 might not bind IAP or PLAP alone, but bind the heterodimer of them.

Therefore, we further transferred the plasmids of IAP, PLAP, or both of them to PC‐3 cells (BG2‐nonbinding cells). As shown in Figure 3b, BG2 did not bind the PC3 cells overexpressed IAP or PLAP alone, but bound the PC3 cells overexpressed both IAP and PLAP, suggesting that BG2 bound to the heterodimer of IAP and PLAP. Since GCAP was also identified with high SILAC ratio, we further tested whether BG2 bound GCAP or heterodimer of GCAP and IAP/PLAP by plasmid transfection experiment. GCAP is highly homologous to PLAP with 98% amino acid similarity, and differ by only 10 amino acids,^18^ no commercial available antibody can distinguish PLAP and GCAP; therefore, anti‐PLAP was used to detect the expression of GCAP. As shown in Figure 3c, BG2 did not bind PC3 cells transfected with only GCAP plasmid or both GCAP and PLAP plasmids, but bound to PC‐3 cells transfected with IAP and GCAP plasmids together, suggesting that BG2 bound IAP–GCAP heterodimer, but did not bind PLAP–GCAP heterodimer. All the AP expression on cells was confirmed by western blot assay (Figure 3d).

When we re‐examined the above results of flow cytometry (Figure S10, Supporting Information) and western blot (Figure 2b), we found that the expression of IAP on LoVo cells was much lower than that of PLAP. Interestingly, after being pulled down by BG2, the amount of IAP was comparable with PLAP (Figure 2b). The most likely explanation is that BG2 bound to the IAP–PLAP heterodimer, but not homodimers or monomers of them. In order to obtain the direct evidences of that BG2 bound IAP–PLAP heterodimer, we crosslinked proteins on surface of LoVo cells in situ by disuccinimidyl suberate (DSS), then pulled down proteins with BG2, and analyzed by western blot (the crosslinked proteins by DSS could not be broken under the used denaturation condition for SDS‐PAGE). As shown in Figure 3e, without crosslinking, only IAP and PLAP monomers but no protein dimers were detected in cell lysate, the amount of IAP monomer was much lower than PLAP monomer (lane 1); after crosslinking, IAP, PLAP monomers, and PLAP homodimers were detected in cell lysate (lane 2); after crosslinking and BG2 pulling down, IAP and PLAP monomers and IAP–PLAP heterodimer were detected (no PLAP homodimer was detected), and the amount of IAP monomer was similar with PLAP monomer (lane 3). Because the efficiency of crosslinking was usually low, the uncrosslinked heterodimers pulled down by BG2 appeared in equal amount of IAP and PLAP monomers. This result provided the direct evidence that BG2 bound IAP–PLAP heterodimer.

### 2.4 Application of Aptamer BG2 as a Molecular Probe

**Figure 4:**
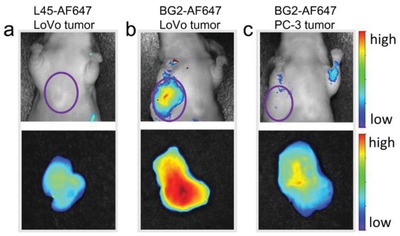
In vivo and ex vivo tumor imaging stained by BG2. LoVo cell xenografted BALB/c nu/nu mice were intravenously injected with a) control sequence L45‐AF647, and b) BG2‐AF647; c) PC‐3 cell xenografted BALB/c nu/nu mouse was intravenously injected with BG2‐AF647. Up: in vivo imaging, down: ex vivo tumor imaging.

Above results also demonstrated that aptamer BG2 could serve as a molecular probe for monitoring the AP heterodimers on cells, and as a ligand for purification. To further prove its feasibility for in vivo applications, tumor imaging in mice xenografted with LoVo (AP heterodimer positive cell) or PC‐3 (AP heterodimer negative cell) cells was performed. As shown in **Figure** **4**, after being intravenously injected with BG2‐AF647, bright fluorescence was observed in tumor‐implanted site of LoVo tumor‐bearing mouse, while only very weak fluorescence was observed in tumor site of PC‐3 tumor‐bearing mouse. In contrast, there was almost no fluorescence detected in LoVo tumor‐bearing mouse injected with L45‐AF647 (control sequence). These results suggest that aptamer BG2 can only recognize tumor highly expressed AP heterodimer in vivo rather than AP monomers or homodimers normally expressed in other tissues. This is the first probe that could detect AP heterodimer in vivo.

## 3 Discussion

As shown above, we have proved that the molecular target of aptamer BG2 is the heterodimers of IAP and PLAP/GCAP. This is the first probe that specifically recognizes a protein dimer. Aptamer BG2 folds into a duplex stabilized G‐quadruplex, and the G‐quadruplex is the essential motif for target binding. G‐quadruplex is a four‐stranded structure of nucleic acid, which is composed of planar G‐quartet formed by four guanine bases stabilized by eight Hoogsteen hydrogen bonds, compared with duplex structure, G‐quadruplex possesses much more variable domains that interact with adjacent molecules, such as grooves, loops, and terminal G‐quartet planes.^19^ Because the size of the G‐quadruplex motif of BG2 is small, it might be specifically recognized by a conserved pocket formed by IAP and PLAP. Because GCAP is highly homologous to PLAP, BG2 also bound IAP–GCAP heterodimer.

AP isozymes, such as PLAP, IAP, and GCAP, are found in variety of tissues, which are detected by corresponding monoclonal antibodies.^3^, ^4^ The general function of APs is to catalyze the hydrolysis of phosphate esters. APs are associated with cell differentiation potential and stemness during development, and are reported as markers for pluripotent embryonic stem cells and cancers.^20^ For example, PLAP or IAP is reported to overexpress in breast cancer, hepatocellular carcinoma, cervical cancer, colon cancer, neoplasia as germ cell tumors, and squamous cell carcinoma of the lung.^17^, ^21^ PLAP is also an effective target for immunotoxin therapy of cancers in vivo using drugs conjugated to PLAP‐specific antibodies.^22^ Polyclonal and monoclonal antibodies against PLAP or GCAP have been used for tumor immunolocalization and immunotherapy of PLAP‐ or GCAP‐producing tumors in both mice and humans. AP homodimers and heterodimers have been found to be present in cancer cells through protein electrophoresis, inhibition by different inhibitors, and immunoassay with two monoclonal antibodies.^4^, ^23^ However, these methods cannot directly prove the formation of AP heterodimers rather than the coexistence of two AP homodimers or monomers on cells. During the last decades, many clinical reports have referred to the relationship of the expression of AP homodimers and malignancy, often with greatly discordant results.^24^ One possibility is that AP heterodimers were mistaken for AP homodimers based on the immunoassay with monoclonal antibodies. Because of the lack of specific probes for heterodimers, currently, it is impossible to know the distribution of AP heterodimers on certain cells and tissues, and thus it is impossible to establish correlation between the heterodimers and diseases (e.g., cancers). In this case, using aptamer BG2 as probes, we found that IAP–PLAP/GCAP heterodimers were specifically expressed on the surface of cell lines derived from colon cancer, breast cancer, cervical cancer, and hepatocellular carcinoma cells by the direct evidence for the first time (Figure 1b). We also used BG2 as an affinity ligand to pull down IAP–PLAP/GCAP heterodimers. Given the advantages of aptamers, BG2 holds the potential as a versatile tool for the biomedical study of AP heterodimer. Further investigation in this area will provide a clear understanding of the correlation between the heterodimers and cancers.

Protein dimerization is a dynamic process occurring in a given cellular environment. The dimers are formed by two protein monomers through noncovalent interactions, and generate new active sites or binding pockets at the dimer interface.^1^ When leaving the cellular environment, the dimers would dissociate or the conformation of their active sites would be changed. Therefore, it is very difficult to obtain a native protein dimer to prepare antibodies or screen other kind of molecular probes. In current case, the cell‐SELEX strategy avoided the isolation of protein dimer and allowed the dimers to be present on the surface of live cells in their active state, which made the discovery of aptamers specifically binding protein dimers possible. Although the discovery of aptamer against the IAP–PLAP heterodimers is an accidental event in this study, it demonstrates that cell‐SELEX is an effective approach for generating aptamers against protein dimer when using cells highly expressed certain protein dimer as target cells.

In summary, we report here a G‐rich DNA aptamer, BG2 derived by cell‐SELEX, that specifically recognizes IAP–PLAP/GCAP heterodimers. BG2 folds into a duplex stabilized antiparallel G‐quadruplex structure, can recognize cultured cells highly expressing AP heterodimers, and can recognize tumor xenografts in nude mice. These results indicate that BG2 hold great promise in the study of AP heterodimers in vitro and in vivo. To our knowledge, this is the first single molecular probe against a heterodimer protein. Our results also show that cell‐SELEX is a feasible way to generate aptameric probes toward protein dimers.

## 4 Experimental Section

*Materials*: All reagents unless otherwise noted were from Sigma‐Aldrich (St. Louis, MO). Heavy lysine ([^13^C_6_, ^15^N_2_]‐l‐lysine) and arginine ([^13^C_6_]‐l‐arginine), 99% in isotopic purity and 98% in chemical purity, were purchased from Silantes GmbH (Andover, Germany). Oligonucleotides were synthesized by Sangon Biotech Co. Ltd. (Shanghai, China). Washing buffer was prepared by adding 5 mmol MgCl_2_ and 4.5 g glucose into 1 L of phosphate buffer saline (PBS) (contain 8 g NaCl, 0.2 g KCl, 3.58 g Na_2_HPO_4_·12H_2_O, 0.272 g KH_2_PO_4_ per 1 L ddH_2_O, pH = 7.4). Binding buffer was prepared by adding 1 mg mL^−1^ bovine serum albumin (BSA, Sigma) and 0.1 mg mL^−1^ herring sperm DNA (Sigma) into washing buffer.

*Cell Culture*: Cell lines: HeLa (human adenocarcinoma cervical cancer), MCF‐7 (human breast cancer), Jurkat (clone E6‐1) (human acute T lymphoblastic leukemia), HEK‐293 (human embryonic kidney), and SH‐SY5Y (human neuroblastoma), were purchased from the Institute of Basic Medical Science at the Chinese Academy of Medical Sciences (Beijing, China). LoVo and HCT‐116 (human colorectal cancer), PC‐3 (prostate cancer), SMMC‐7721, and HepG2 (human liver hepatocellular carcinoma) were purchased from Typical Culture Preservation Commission Cell Bank, Chinese Academy of Sciences (Shanghai, China). Unless otherwise indicated, all cells were cultured in RPMI 1640 medium supplemented with 10% fetal bovine serum (FBS, Invitrogen) and 100 IU mL^−1^ penicillin‐streptomycin (Invitrogen). Cells were maintained at 37 °C in a humidified atmosphere containing 5% CO_2_, with medium renewal at every 2–3 days. For stable isotope labeling with amino acids in cell culture experiments, the RPMI 1640 medium without l‐lysine or l‐arginine was custom‐prepared following the ATCC formulation. The complete light and heavy RPMI 1640 media were prepared by the addition of light or heavy lysine and arginine, together with dialyzed FBS (PAN‐Biotech Gmbh, Germany), to the above lysine, arginine‐depleted medium. LoVo cells were cultured in light or heavy RPMI 1640 medium for at least six cell doublings to achieve complete isotope incorporation.

*Selection of Cell Binding Aptamers*: DNA library, Blib: 5′‐ACGCTC GGATGCC ACTACAGTYRRRRRRNN*GGGNNNGG*NNN*GGNNGG*NNNNNNNN GGNYYYYYYRTCTCATGGACGTGCTGGTGAC‐3′, where N = A, T, G or C; Y = C or T; R = A or G. Forward primer, BP‐1:5′‐FAM‐ACGCTCGGATGCCACTACAG‐3′; Reverse primer, BP2:5′‐ biotin‐GTCACCAGCACGTCC ATG AG ‐3′. HeLa was used as target cell line. The cell‐SELEX protocol was similar to that described previously.^13^ Briefly, ssDNA library (12 nmol) was dissolved in 500 µL of binding buffer and denatured by heating at 95 °C for 5 min and cooled on ice for 5 min before usage. HeLa cells with 75–90% confluence were dissociated by PBS containing 5 × 10^−3^ m EDTA, and then washed with washing buffer once. The library was incubated with HeLa cells at 4 °C for 30 min, and then cells were washed twice by cold washing buffer. The bound DNA was eluted by adding 200 µL of ddH_2_O, and heating at 95 °C for 5 min. After centrifugation, the supernatant was used as template and amplified by PCR with the primers (8–12 cycles of 30 s at 94 °C, 30 s at 60 °C, and 30 s at 72 °C, followed by 3 min at 72 °C). The FAM‐labeled ssDNA pool was separated from the PCR product by streptavidin‐coated sepharose beads and NaOH denaturation for the next round selection. The number of cells used in the selection decreased in each round, approximately from 5 × 10^6^ to 1 × 10^6^. The selection process was monitored using flow cytometry. After five rounds of selection, the enriched DNA pool was sequenced using high throughput sequencing by Sangon Biotechnology Co., Ltd.

*Circular Dichroism Analysis*: Circular dichroism analysis was performed on a JASCO spectrometer with 10 mm path length quartz cuvettes. The spectra measurements were carried out with 100 nm min^−1^, at a 2 nm bandwidth, at 25 °C and normalized by subtraction of the background scan of the buffer.

*Flow Cytometric Analysis*: A total of 5 × 10^5^ cells were incubated with antibodies (anti‐IAP, GTX60746, Gene Tex; anti‐PLAP, MA1‐20245, Thermo Fisher Scientific) and/or FITC‐ or AF647‐labeled aptamers in 100 µL of binding buffer (100 × 10^−9^ m) on ice for 30 min. The cells were washed once, and added with antimouse IgG‐PE. After 30 min incubation, the cells were washed again, resuspended in 0.4 mL of the above‐mentioned binding buffer, and subjected to flow cytometry analysis on a BD FACSCalibur instrument (BD Biosciences). 10 000 events were measured for each cell sample. The FITC‐ or AF647‐labeled, unenriched ssDNA library, and IgG were employed as negative controls. The Pearson's correlation coefficient for the correlation between the aptamer BG2 and anti‐IAP or PALP was calculated with the fluorescence intensity of cells using SPSS 16.0 (SPSS Inc.).

*Cell Binding Assay*: LoVo cells in logarithmic phase were dissociated with PBS containing 5 × 10^−3^ m EDTA and harvested as monodispersed cell suspension. After being washed once and resuspended in binding buffer, cell suspension was incubated with different concentrations of aptamers for 30 min at 4 °C. After incubation, the cells were washed and resuspended in 300 µL of binding buffer. The fluorescence intensity of cells was recorded by flow cytometry. The equilibrium dissociation constants (*K*_d_s) of the aptamer–cell interaction were obtained by fitting the dependence of the mean fluorescence intensity of specific binding on the concentration of the aptamers to the equation Y = B_max_X/(*K*_d_ + X), using GraphPad Prism 6 (San Diego, CA).

*Target Protein Identification by SILAC‐Based Quantitative Proteomic Analysis*: SILAC‐based quantitative proteomic method was used to identify the target protein of aptamer BG2. The detailed procedures and data processing were referred to the protocol previously reported.^14^ Briefly, biotin‐labeled aptamer BG2 or control sequence BC1 was incubated with 2 × 10^8^ heavy‐ or light‐labeled LoVo cells in 4 mL of binding buffer on ice for 30 min, and then 4 mL of PBS buffer containing 2% formaldehyde were added to crosslink BG2 and its target protein in situ. The mixture was incubated on ice for 15 min. Then the cells were lysed and the aptamer‐target complexes were pulled down using streptavidin‐coated beads. In the forward SILAC experiment, the light‐ and heavy‐labeled cells were crosslinked to control sequence BC1 and aptamer BG2, respectively. After being lysed, the protein–DNA complexes were captured by beads from the cell lysates separately and subsequently mixed. On the other hand, in the reverse SILAC experiment, the light‐ and heavy‐labeled cells were crosslinked with BG2 and BC1, respectively, and then the protein–DNA complexes were captured and mixed. Then, the captured proteins were released from the beads by reducing SDS loading buffer and run on a 12% SDS‐PAGE at 150 V for 15 min to about 9–10 mm. The proteins were digested in‐gel with trypsin. The resulting peptides were collected, dried in a Speed‐vac, and stored at −20 °C until LC‐MS and MS/MS analysis. The MS experiments were performed on an Orbitrap Fusion mass spectrometer with a nanoelectrospray ionization source (Thermo Fisher Scientific, San Jose, CA). The original mass spectral data was searched using the MaxQuant search engine (version number: 1.5.5.1) in the UniProt database. The intensity ratios for heavy/light‐labeled peptides of the candidate aptamer targets were further validated by manual analysis, where the intensity ratios were taken across the peaks found in the selected‐ion chromatograms for precursor ions of the unique peptides derived from the candidate aptamer targets. The intensity ratios for light/heavy‐labeled peptides are calculated as mean ±S.D. using Origin 8.0 Software (Microcal Software, Northampton, MA) according to two forward and reverse SILAC labeling experiments (*n* = 4).

*The Activity Detection of APs Pulled Down by Aptamer*: Biotin‐labeled aptamer BG2 or control sequence were incubated with 1 × 10^7^ LoVo cells on ice for 30 min in 0.4 mL of binding buffer, after which 0.4 mL of PBS buffer containing 2% formaldehyde were added. The mixture was incubated on ice for 15 min to induce crosslinking in situ. Then the cells were lysed and the aptamer‐target complexes were pulled down using streptavidin‐coated beads. The AP activity of the pull‐down protein was measured using an Alkaline Phosphatase Assay Kit (Beyotime, China) according to the manufacturer's instruction.

*Western Blot Analysis of Pull‐Down APs by Aptamer*: Rabbit monoclonal antibodies against PLAP (ab133602) and IAP (ab186422) (1:5000 dilution, Abcam) were used for western blot analysis. Membrane proteins were extracted using Mem‐PER Plus Membrane Protein Extraction Kit (Thermo Fisher Scientific) according to the protocol. 2.5 µL of membrane proteins or aptamer‐pulled‐down proteins were mixed with 2.5 µL of 4× SDS sample loading buffer with β‐mercaptoethanol (Bio‐Rad) and heated at 100 °C for 30 min, and then the samples were separated by 6% SDS‐PAGE with a 5% stacking gel. After gel separation, proteins were transferred to a PVDF membrane (Millipore, USA). The membranes were blocked with 5% nonfat milk (Sangon, China) in PBS buffer containing 0.1% v/v Tween‐20 (PBST, pH 7.5) for 1 h at room temperature, and incubated overnight at 4 °C with corresponding antibodies. The membrane was washed with fresh PBST at room temperature for five times following incubation with HRP‐conjugated secondary antibody (1:5000 dilution, Santa) at room temperature for 1 h. The membranes were subsequently washed with PBST for five times and detected using SuperSignal West Femto Maximum Sensitivity Substrate (Thermo Fisher Scientific).

*Cell Surface Anchoring of PLAP Proteins*: For cell surface anchoring with GPI‐linked PLAP protein, the Jurkat cells (AP nonexpressing) were washed twice, resuspended in serum‐free medium at 2 × 10^6^ cells mL^−1^, and incubated with human PLAP protein (0.2 mg mL^−1^) for 2 h at 37 °C for occasional inversion. Anchored cells were extensively washed, and the membrane insertion efficiency assessed by FACS analysis.

*APs or L1CAM Knock Down with siRNA*: Small interfering RNA targeting IAP, PLAP, or L1CAM gene and siRNA universal negative control (Genepharma) that does not target any human gene product were synthesized by Genepharma. siRNA transfection was carried out using Lipofectamine RNAiMAX Transfection Reagent (Thermo Scientific). Aptamer or antibody binding was examined at 72 h post siRNA treatment by flow cytometry.

*Transfection of APs in PC‐3 Cells*: The cDNA of IAP (P09923) or PLAP (P05187) constructed by PCR‐based amplification of cDNA from LoVo cells was inserted into mammalian expression plasmid pCMV‐myc vector with restriction endonucleases (Xho1 and EcoR1, NEB), respectively. The cDNA of GCAP (P10696) purchased from Youbio Biological Technology Co., Ltd. was inserted into mammalian expression plasmid pcDNA3.1(‐) plasmid with restriction endonucleases (Xho1 and Bamh1). These plasmids were transfected into PC‐3 cells in a 6‐well plate using Lipofectamine 3000 Transfection Reagent (Thermo Scientific) with 3 µg of plasmid and 6 µL of transfection reagent per well. After 48 h of transfection, cells were collected and stained with aptamers or antibodies as above.

*Confocal Image of Cells*: 2 × 10^4^ cells were cultured in covered glass‐bottomed cell confocal dishes (NEST Biotechnology Co. Ltd.) for more than 1 day to get a well extension. For living cell imaging, cells were incubated with aptamers at 4 °C for 30 min. For fixed cell imaging, cells were fixed using 1% formaldehyde in PBS on ice for 10 min, and then incubated with aptamers at 4 °C for 30 min. Confocal microscopy imaging was performed with an OLYMPUS FV1000‐IX81 confocal microscope (Olympus Corporation, Japan) with different objective lens. The images were analyzed by FV10‐ASW.

*Western Blot Assay of Protein Dimers*: 1 × 10^7^ LoVo cells were incubated with or without Biotin‐disulfide‐labeled aptamer BG2 on ice for 30 min in 0.5 mL of binding buffer, after which 25 µL of 100 × 10^−3^ m disuccinimidyl suberate (Thermo Fisher Scientific) were added to crosslink the protein dimers. The mixture was incubated on ice for 120 min to induce crosslinking in situ and terminated by the addition of 25 µL of 1 m Tris‐HCl buffer, pH 7.0. Then, cells were lysed and the aptamer‐target complexes were pulled down using streptavidin‐coated beads. The membrane proteins or the aptamer‐pulled‐down proteins were mixed with 2.5 µL of 4× SDS sample loading buffer with β‐mercaptoethanol (Bio‐Rad) and heated at 60 °C for 10 min, and then the samples were separated by 6% SDS‐PAGE with a 5% stacking gel; the western blot assay of the pull‐down proteins was performed as above.

*In Vivo and Ex Vivo Optical Imaging*: All animal experiments were performed in strict compliance with the guide of the Care and Use of Laboratory Animals of Department of Laboratory Animal Science, Peking University Health Science Center. Prior to initiation of the experiments, mice were acclimatized to husbandry conditions for 1 week to eliminate the stress. 4–6 weeks old female BALB/c nu/nu mice (obtained from Beijing Vital River Laboratory Animal Technology Co., Ltd.) received a subcutaneous injection of 3 × 10^6^ LoVo or PC‐3 cancer cells into right armpit. Tumors were then allowed to grow to 0.6–1.2 cm in diameter for about 4 weeks. Before imaging, BALB/c nu/nu mice were anesthetized with the combined use of tranquilizer and anesthetic. Once the mice were anesthetized to be motionless, a 100 µL of BG2‐AF647 (3 µM) or random sequence L45‐AF647 (3 µM) was injected intravenously via the tail vein. After 30 min, fluorescence images of chest side of live mice were taken by a Maestro in vivo fluorescence imaging system (Cambridge Research & Instrumentation, Inc.). A 586–601 nm bandpass filter and a 640 nm longpass filter were selected to be used as the excitation filter and the emission filter, respectively. For ex vivo imaging, excised tumors were placed in the optical imaging system and imaged as above. The fluorescence images were presented after processing by Maestro software (version 2.10.0).

## Conflict of Interest

The authors declare no conflict of interest.

## Supporting information

Supplementary

## References

[1] a) N. J. Marianayagam , M. Sunde , J. M. Matthews , Trends Biochem. Sci. 2004, 29, 618; 15501681 10.1016/j.tibs.2004.09.006

[2] a) H. Guo , S. An , R. Ward , Y. Yang , Y. Liu , X. X. Guo , Q. Hao , T. R. Xu , Biosci. Rep. 2017, 37, BSR20160547; 28062602 10.1042/BSR20160547 PMC5398257

[3] H. Imanishi , T. Hada , K. Muratani , K. Hirano , K. Higashino , Cancer Res. 1990, 50, 3408. 2334935

[4] a) H. Kodama , K. Asai , T. Adachi , Y. Mori , K. Hayashi , K. Hirano , T. Stigbrand , Biochim. Biophys. Acta, Gene Struct. Expression 1994, 1218, 163; 10.1016/0167-4781(94)90006-x 8018716

[5] A. Wada , A. P. Wang , H. Isomoto , Y. Satomi , T. Takao , A. Takahashi , S. Awata , T. Nomura , Y. Fujii , S. Kohno , K. Okamoto , J. Moss , J. L. Millan , T. Hirayama , Int. J. Med. Microbiol. 2005, 294, 427. 15715171 10.1016/j.ijmm.2004.09.012

[6] a) W. Zhang , Y. Jiang , Q. Wang , X. Ma , Z. Xiao , W. Zuo , X. Fang , Y. G. Chen , Proc. Natl. Acad. Sci. USA 2009, 106, 15679; 19720988 10.1073/pnas.0908279106 PMC2747179

[7] a) M. Gullberg , A.‐C. Andersson , Nat. Methods 2010, 7, 10;

[8] S. Buus , J. Rockberg , B. Forsstrom , P. Nilsson , M. Uhlen , C. Schafer‐Nielsen , Mol. Cell. Proteomics 2012, 11, 1790. 22984286 10.1074/mcp.M112.020800 PMC3518105

[9] a) A. D. Ellington , J. W. Szostak , Nature 1990, 346, 818; 1697402 10.1038/346818a0

[10] F. Opazo , M. Levy , M. Byrom , C. Schafer , C. Geisler , T. W. Groemer , A. D. Ellington , S. O. Rizzoli , Nat. Methods 2012, 9, 938. 23018995 10.1038/nmeth.2179

[11] B. Deng , Y. W. Lin , C. Wang , F. Li , Z. X. Wang , H. Q. Zhang , X. F. Li , X. C. Le , Anal. Chim. Acta 2014, 837, 1. 25000852 10.1016/j.aca.2014.04.055

[12] a) L. Q. Zhang , S. Wan , Y. Jiang , Y. Y. Wang , T. Fu , Q. L. Liu , Z. J. Cao , L. P. Qiu , W. H. Tan , J. Am. Chem. Soc. 2017, 139, 2532; 28121431 10.1021/jacs.6b10646 PMC5519284

[13] a) N. Zhang , T. Bing , L. Y. Shen , R. S. Song , L. L. Wang , X. J. Liu , M. R. Liu , J. Li , W. H. Tan , D. H. Shangguan , Angew. Chem., Int. Ed. 2016, 55, 3914; 10.1002/anie.201510786 26889661

[14] T. Bing , D. Shangguan , Y. Wang , Mol. Cell. Proteomics 2015, 14, 2692. 26199357 10.1074/mcp.M115.051243 PMC4597145

[15] M. Wilhelm , J. Schlegl , H. Hahne , A. M. Gholami , M. Lieberenz , M. M. Savitski , E. Ziegler , L. Butzmann , S. Gessulat , H. Marx , T. Mathieson , S. Lemeer , K. Schnatbaum , U. Reimer , H. Wenschuh , M. Mollenhauer , J. Slotta‐Huspenina , J. H. Boese , M. Bantscheff , A. Gerstmair , F. Faerber , B. Kuster , Nature 2014, 509, 582. 24870543 10.1038/nature13319

[16] a) H. Kohno , K. Sudo , T. Kanno , Clin. Chim. Acta 1983, 135, 41; 6418416 10.1016/0009-8981(83)90386-8

[17] U. Sharma , D. Pal , R. Prasad , Indian J. Clin. Biochem. 2014, 29, 269. 24966474 10.1007/s12291-013-0408-y PMC4062654

[18] M. H. Le Du , T. Stigbrand , M. J. Taussig , A. Menez , E. A. Stura , J. Biol. Chem. 2001, 276, 9158. 11124260 10.1074/jbc.M009250200

[19] E. Ruggiero , S. N. Richter , Nucleic Acids Res. 2018, 46, 3270. 29554280 10.1093/nar/gky187 PMC5909458

[20] a) M. D. O'Connor , M. D. Kardel , I. Iosfina , D. Youssef , M. Lu , M. M. Li , S. Vercauteren , A. Nagy , C. J. Eaves , Stem Cells 2008, 26, 1109; 18276800 10.1634/stemcells.2007-0801

[21] a) L. C. Tsai , M. W. Hung , Y. H. Chen , W. C. Su , G. G. Chang , T. C. Chang , Eur. J. Biochem. 2000, 267, 1330; 10691970 10.1046/j.1432-1327.2000.01100.x

[22] K. Tsukazaki , E. G. Hayman , E. Ruoslahti , Cancer Res. 1985, 45, 1834. 2579736

[23] H. Imanishi , T. Hada , K. Muratani , K. Hirano , K. Higashino , Clin. Chim. Acta 1990, 186, 309. 2311258 10.1016/0009-8981(90)90049-x

[24] J. L. Millán , Mammalian alkaline phosphatases: from biology to applications in medicine and biotechnology, Wiley‐VCH Verlag GmbH & Co. KGaA, Weinheim, Germany 2016, p. 187.

