# Supplementary material for: Aptameric Probe Specifically Binding Protein Heterodimer Rather Than Monomers
Source: Adv Sci (Weinh). 2019 Apr 9;6(11):1900143. doi: 10.1002/advs.201900143 (PMC6548965; doi:10.1002/advs.201900143)
Supplement: Supplementary file 1 — Supplementary [file ADVS-6-1900143-s001.pdf]

Copyright WILEY-VCH Verlag GmbH & Co. KGaA, 69469 Weinheim, Germany, 2019.

ADVANCED SCIENCE
Open Access

Supporting Information

for *Adv. Sci.,* DOI: 10.1002/advs.201900143

Aptameric Probe Specifically Binding Protein Heterodimer Rather Than Monomers

*Tao Bing, Luyao Shen, Junyan Wang, Linlin Wang, Xiangjun Liu, Nan Zhang, Xiao Xiao, and Dihua Shangguan**

Supplementary Information for

Aptameric probe specifically binding protein heterodimer rather than monomers

Tao Bing[1, 2, †], Luyao Shen[1, 2, †], Junyan Wang[1, 2], Linlin Wang[1, 2], Xiangjun Liu[1, 2], Nan Zhang[1,2] , Xiao Xiao[1, 2] and Dihua Shangguan[1, 2, *]

† These authors contributed equally to this work.

[*] To whom correspondence should be addressed. Email: sgdh@iccas.ac.cn

Supplementary Results and Discussion

Cell-SELEX. Usually, the specific binding of aptamers results from their tertiary structures composed of secondary structures, such as hairpins, bulges, pseudoknots, T-junctions, G-quadruplexes, and triplex-quadruplex [1, 2]. Previously, we have generated a series of aptamers against small molecules, peptides, proteins and cells [2, 3, 4]. Many of these aptamers are found to adopt a hairpin structure with a guanine(G)-rich loop predicted by mFold software [5]. The G-rich loops are further demonstrated to form G-quadruplex structures, and the stems usually play the role of stabilizing G-quadruplex structures [3, 6], suggesting that the duplex stabilized G-quadruplex structure is a favored aptamer binding structure. Szostak et al. have reported that incorporating a specific stem-loop into a random library increases the functional potential of the library [7]. David R. Liu et al. have reported a patterned library with alternating pyrimidine and purine rich positions to favor the formation of stem-loops and bulges, which significantly increases the efficiency of SELEX [8]. Therefore, we designed a restricted library that is favorable to form the duplex stabilized G-quadruplex instead of a totally random library.

Based on this idea, we designed a restricted DNA library, 5'-ACGCTCGGATGCCACTACAGTYRRRRRRNNGGGNNNGGNNNGGNNGGNNNNNNNNGGNYYY YYYRTCTCATGGACGTGCTGGTGAC-3. In this library, a YRRRRRR and a YYYYYYR sequences (Y = C or T; R = A or G) were set within the random region next to the primer binding regions to increase the probability of forming a 7-bp stem; 5 G-tracts were placed in the central 30-bp regions to increase the probability of forming G-quadruplex; and other bases (N) were totally random. The SELEX was performed using a human cervical denocarcinoma cell line, HeLa, as target cells. The aptamer enrichment was monitored by flow cytometry. After three rounds of selection, the fluorescence intensity of cells was greatly increased; and almost no much fluorescence increase was observed after two further rounds of selection (Figure S1a), suggesting that aptamers were enriched in three rounds. Compared with our previous aptamer selections those spent about 20 rounds,[9] the efficiency of SELEX was greatly improved by using the restricted library. The enriched pool also found to bind MCF-7 and LoVo cells (Figure S1b and c).

The enriched DNA pool of the fifth round was sequenced by high throughput sequencing on an Illumina platform (Sangon Biotech Co. Ltd). About 15000 tests were sequenced, which contained 6628 unique sequences. Almost all sequences contained two complete complementary sequences near the primer regions, which could form the stem of hairpin structure, and 42% of them contained a conserved G-rich motif, GGGGTCGGTGTGGGTGGTTATGATTGG, suggesting that it may be the binding motif of aptamers.

The binding property of aptamer BG2. Many cell binding aptamers are selected at 4 ^{o}C or on ice; they usually showed weaker binding capability at 37 ^{o}C, which could hinder their applications in physiological conditions.[10] The binding assays showed that BG2 exhibited similar binding ability to LoVo cells at 4

°C,25 °C and 37 °C (Figure 2Sa), while the non-stem sequence, BG2c showed slightly weaker binding capability at 25 °C and 37 °C than at 4 °C (Figure S2b), suggesting that the stem of BG2 stabilized its binding structure and made it work well at 37 °C. In addition, BG2 maintained good binding ability to LoVo cells after incubation at 37 °C in culture medium containing 10% FBS for 30 min, 1 hour and 3 hours (Figure S2c). These results suggest that BG2 has the potential for in vivo usage.

The proteinase treatment experiment showed that treating cells with trypsin or proteinase K did not affect the binding of BG2 (Figure S2d), suggesting that the molecular target of BG2 on cell surface may be a proteinase-resistant protein, a protein present in a membrane domain that are inaccessible to proteinase, or even not a protein.

Many antibodies and aptamers bind living cells but do not bind formalin-fixed cells or tissues.[11] The flow cytometry assay and confocal imaging showed that after fixed LoVo cells with 1% formaldehyde, BG2 still bound the fixed cells with similar affinity to living cells (Figure S2e and f). This finding suggests that aptamer BG2 has the potential as a molecular probe for the analysis of live cells, fixed cells and tissue sections.

The AP antibodies. It should be noticed that there was cross-reaction between monoclonal antibodies against IAP, PLAP and GCAP because of the 85% homology between IAP and PLAP, and 98% homology between PLAP and GCAP. Four commercially available monoclonal antibodies were used in this study. Anti-PLAP (ab133602) and anti-IAP (ab186422) were used for western blot assay, in which anti-PLAP (ab133602) showed weak cross reaction to IAP; but anti-IAP (ab186422) bound both PLAP and IAP. Anti-PLAP (MA1-20245) and anti-IAP (GTX60746) were used for flow cytometric assay and cell imaging; both of them showed weak cross reaction between IAP and PLAP, but anti-IAP (GTX60746) also showed strong cross-reaction to PLAP when used in western blot assay. There was no commercially available anti-GCAP antibody, but the two anti-PLAP antibodies could bind GCAP.

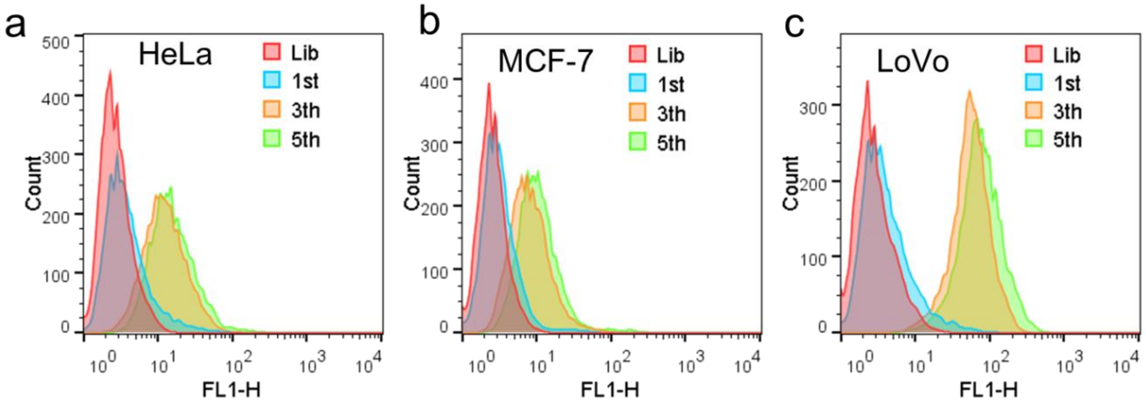

Figure S1. The characterization of enriched pool to different cell lines. The binding of enriched pool to HeLa (a), MCF-7(b) and LoVo (c) cells.

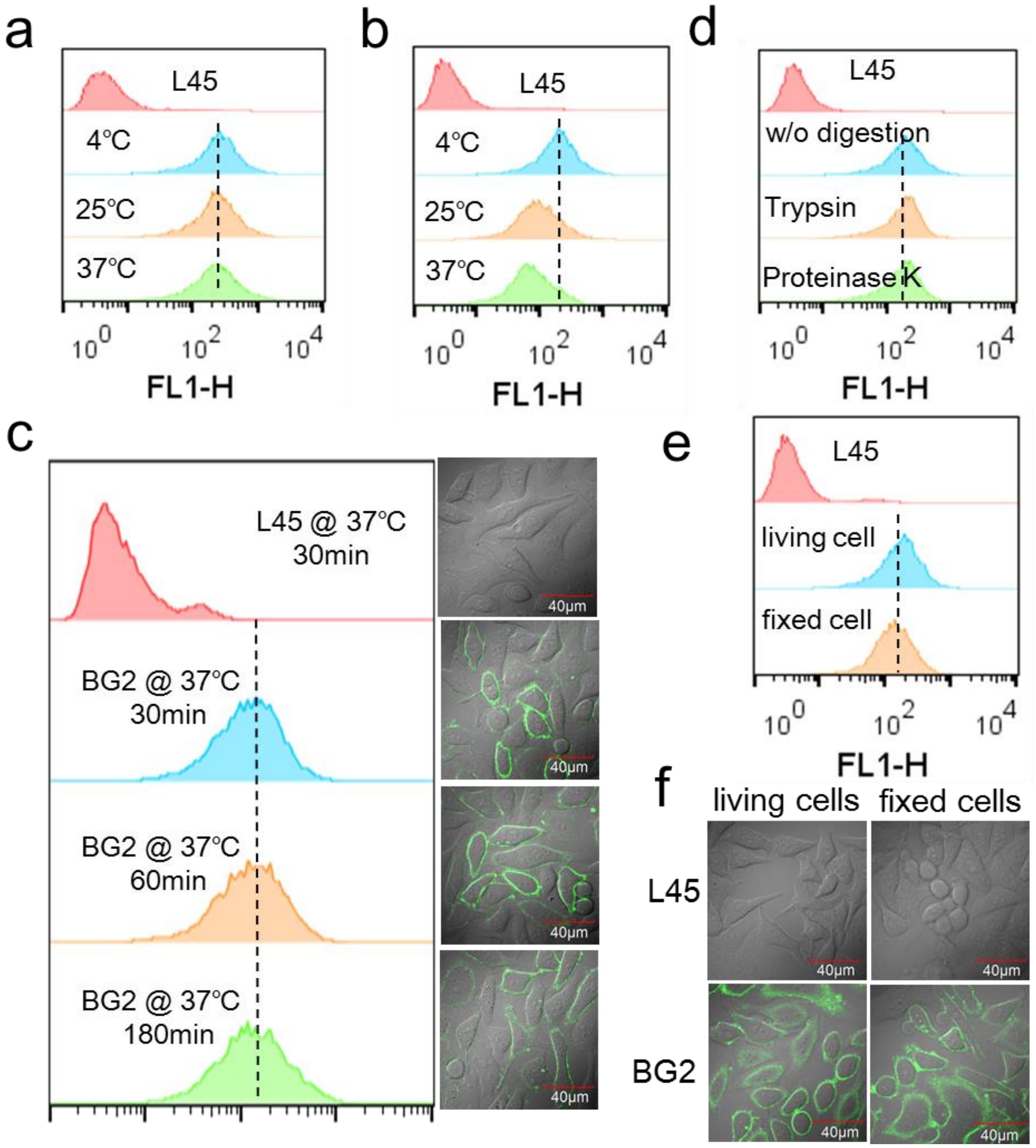

Figure S2. The binding properties of BG2. (a) Effect of incubation temperature on binding ability of BG2. (b) Effect of incubation temperature on binding ability of BG2c. (c) Flow cytometry assay and Fluorescence imaging of LoVo cells after incubation with FAM-labeled library or BG2 (200 nM) at 37 °C in cell culture medium with 10% FBS and 0.1 mg/mL sperm DNA for 30 min, 1 h and 3 h. (d) Effect of proteinase treatment on the binding of BG2, LoVo cells were treated with either 0.05% trypsin or 0.1 μg/μL Proteinase K in PBS at 37 °C for 5 min. (e) Binding of BG2 to living or formaldehyde fixed LoVo cells. (f) Fluorescence imaging of living and formaldehyde fixed LoVo cells stained by BG2. L45, FITC-labeled random sequences used as control.

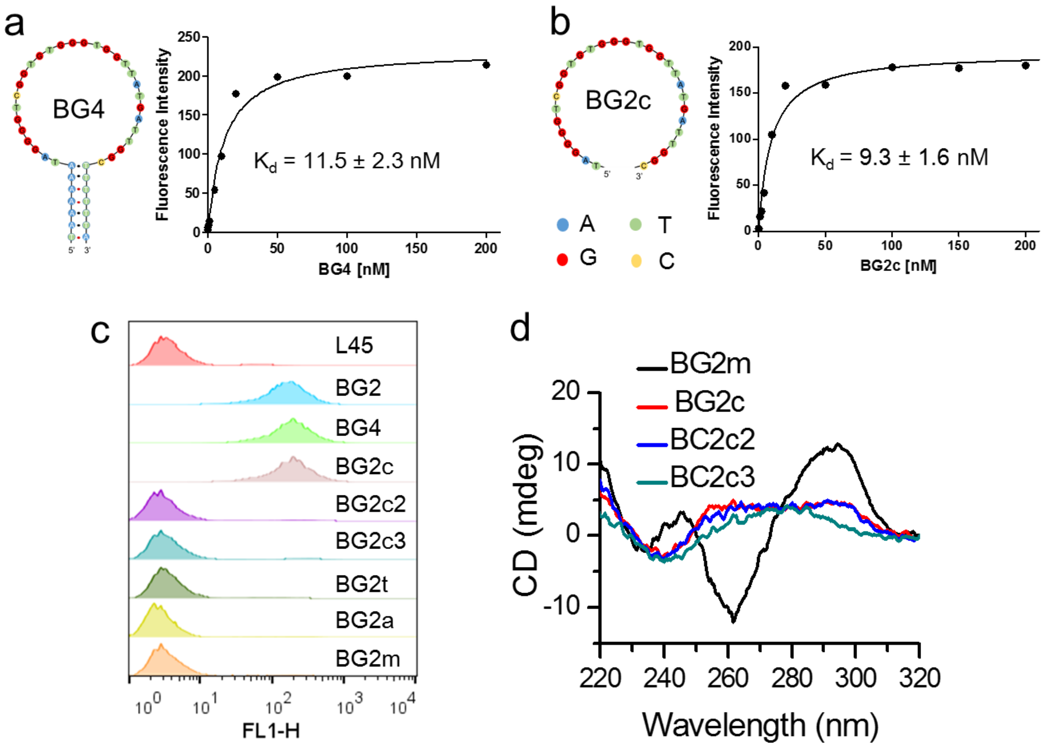

Figure S3. The characterization of derivative sequences of BG2. The apparent equilibrium dissociation constants (K_ds) of BG4 (a) and BG2c (b) to LoVo cells determined by flow cytometry. (c) The binding of mutated sequences of BG-2 to LoVo cells. (d) CD spectra of mutated sequences of BG-2.

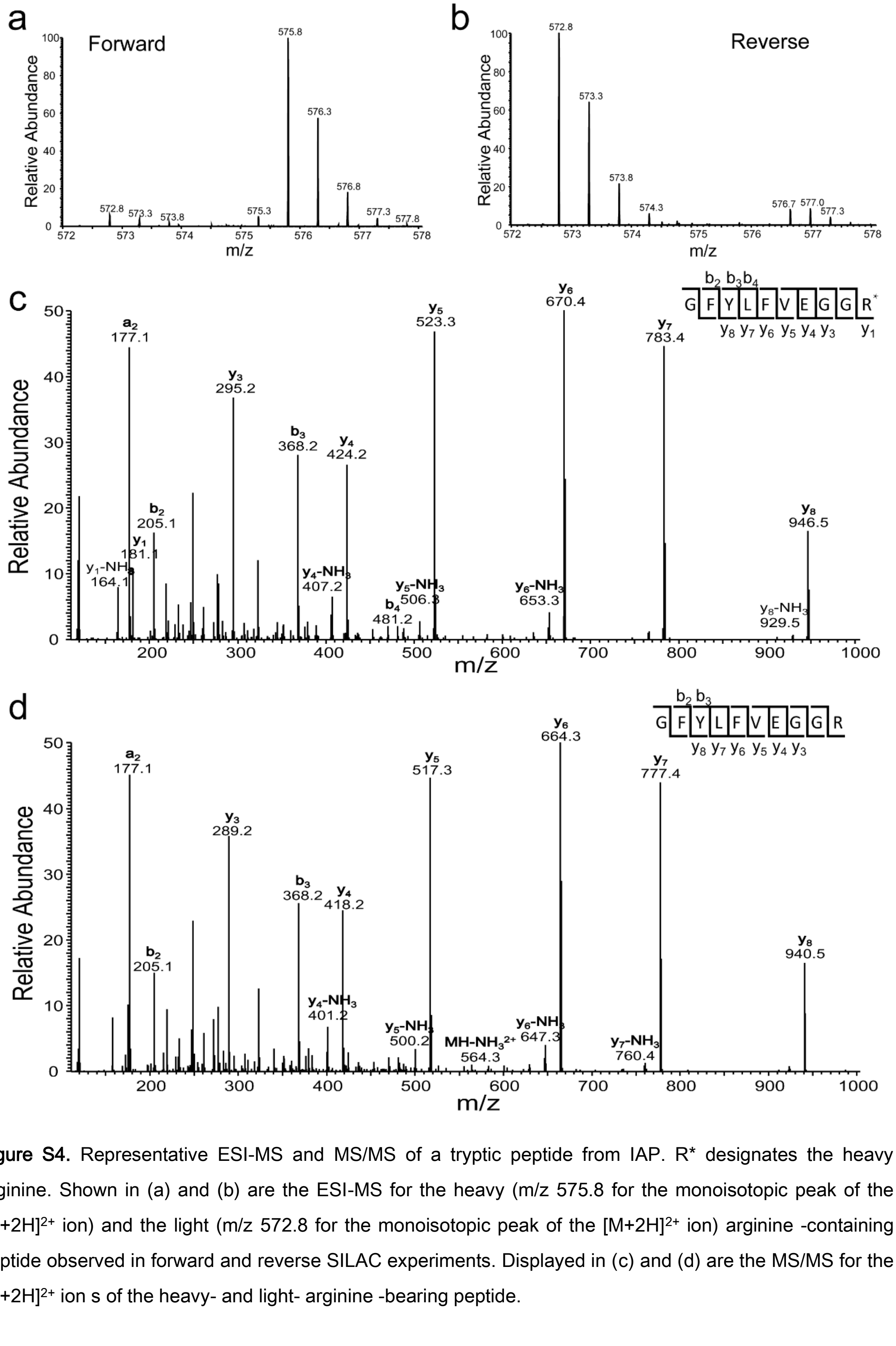

Figure S4. Representative ESI-MS and MS/MS of a tryptic peptide from IAP. R* designates the heavy arginine. Shown in (a) and (b) are the ESI-MS for the heavy (m/z 575.8 for the monoisotopic peak of the $[M+2H]^{2+}$ ion) and the light (m/z 572.8 for the monoisotopic peak of the $[M+2H]^{2+}$ ion) arginine -containing peptide observed in forward and reverse SILAC experiments. Displayed in (c) and (d) are the MS/MS for the $[M+2H]^{2+}$ ion s of the heavy- and light- arginine -bearing peptide.

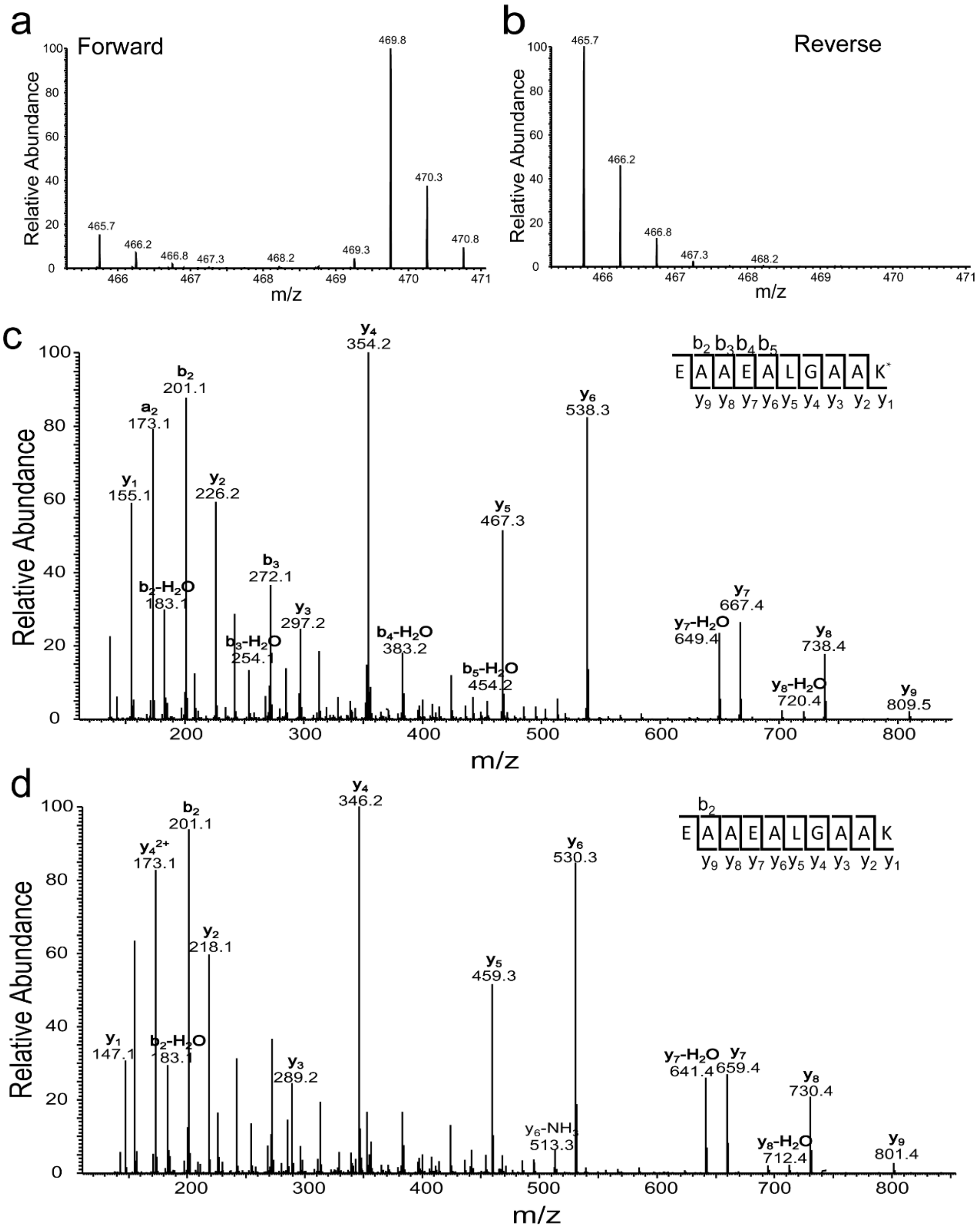

Figure S5. Representative ESI-MS and MS/MS of a tryptic peptide from ALPP. K* designates the heavy lysine. Shown in (a) and (b) are the ESI-MS for the heavy (m/z 469.8 for the monoisotopic peak of the $[M+2H]^{2+}$ ion) and the light (m/z 465.7 for the monoisotopic peak of the $[M+2H]^{2+}$ ion) lysine-containing peptide observed in forward and reverse SILAC experiments. Displayed in (c) and (d) are the MS/MS for the $[M+2H]^{2+}$ ion s of the heavy- and light-lysine-bearing peptide.

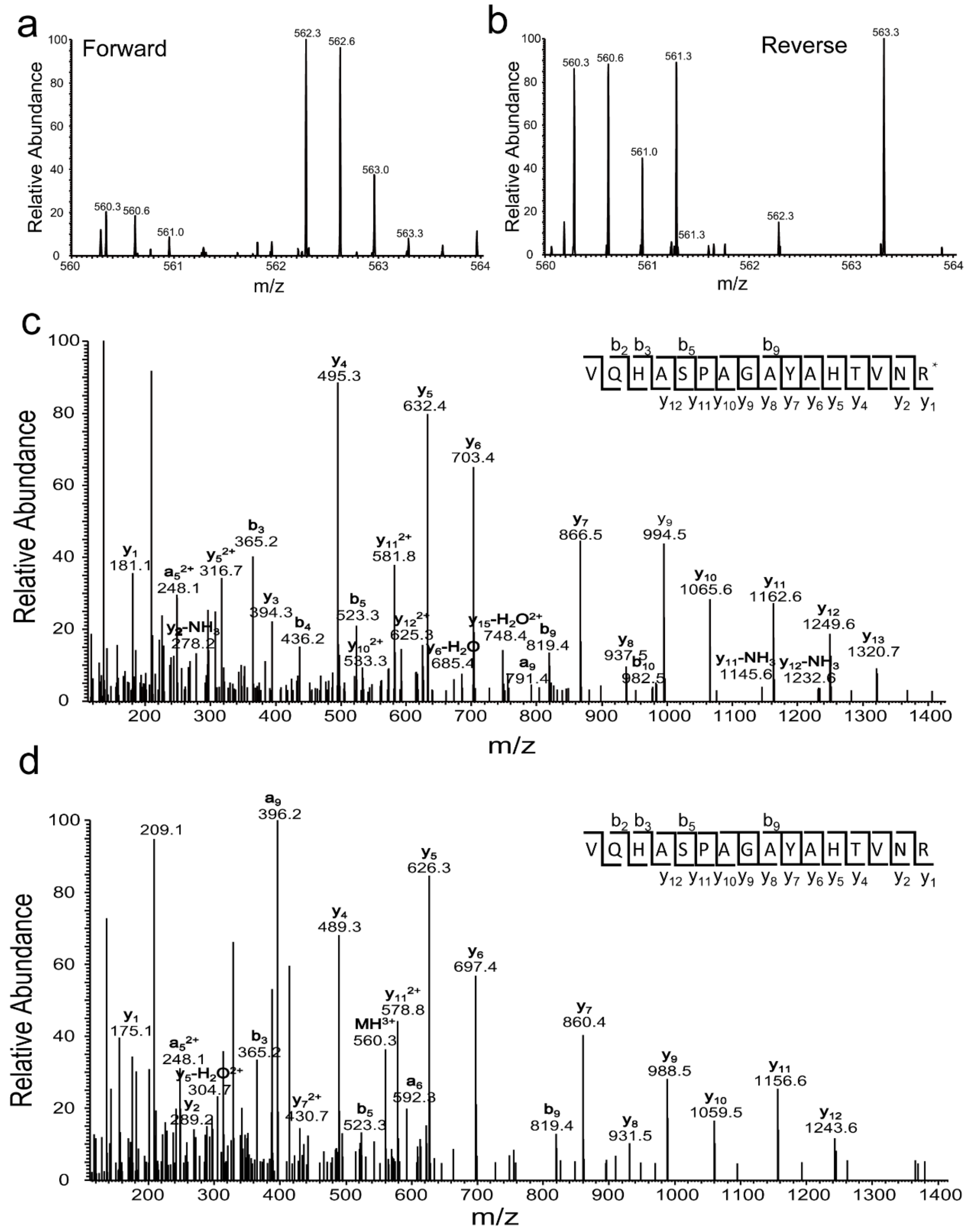

Figure S6. Representative ESI-MS and MS/MS of a tryptic peptide from ALPPL2. R* designates the heavy arginine. Shown in (a) and (b) are the ESI-MS for the heavy (m/z 562.3 for the monoisotopic peak of the $[M+3H]^{3+}$ ion) and the light (m/z 560.3 for the monoisotopic peak of the $[M+3H]^{3+}$ ion) arginine -containing peptide observed in forward and reverse SILAC experiments. Displayed in (c) and (d) are the MS/MS for the $[M+3H]^{3+}$ ion s of the heavy- and light- arginine -bearing peptide.

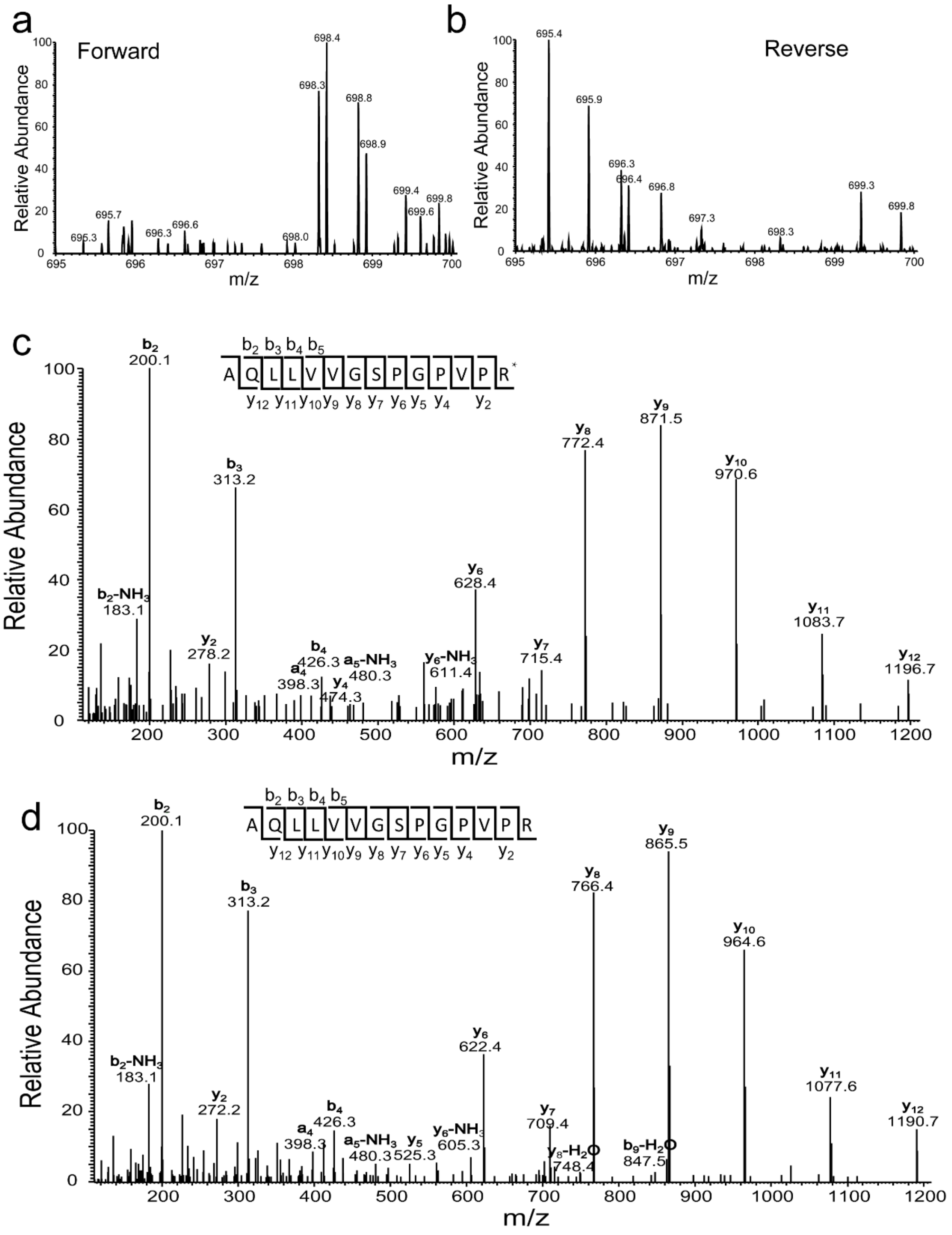

Figure S7. Representative ESI-MS and MS/MS of a tryptic peptide fromL1CAM. R* designates the heavy arginine. Shown in (a) and (b) are the ESI-MS for the heavy (m/z 698.4 for the monoisotopic peak of the $[M+2H]^{2+}$ ion) and the light (m/z 695.4 for the monoisotopic peak of the $[M+2H]^{2+}$ ion) arginine -containing peptide observed in forward and reverse SILAC experiments. Displayed in (c) and (d) are the MS/MS for the $[M+2H]^{2+}$ ion s of the heavy- and light- arginine -bearing peptide.

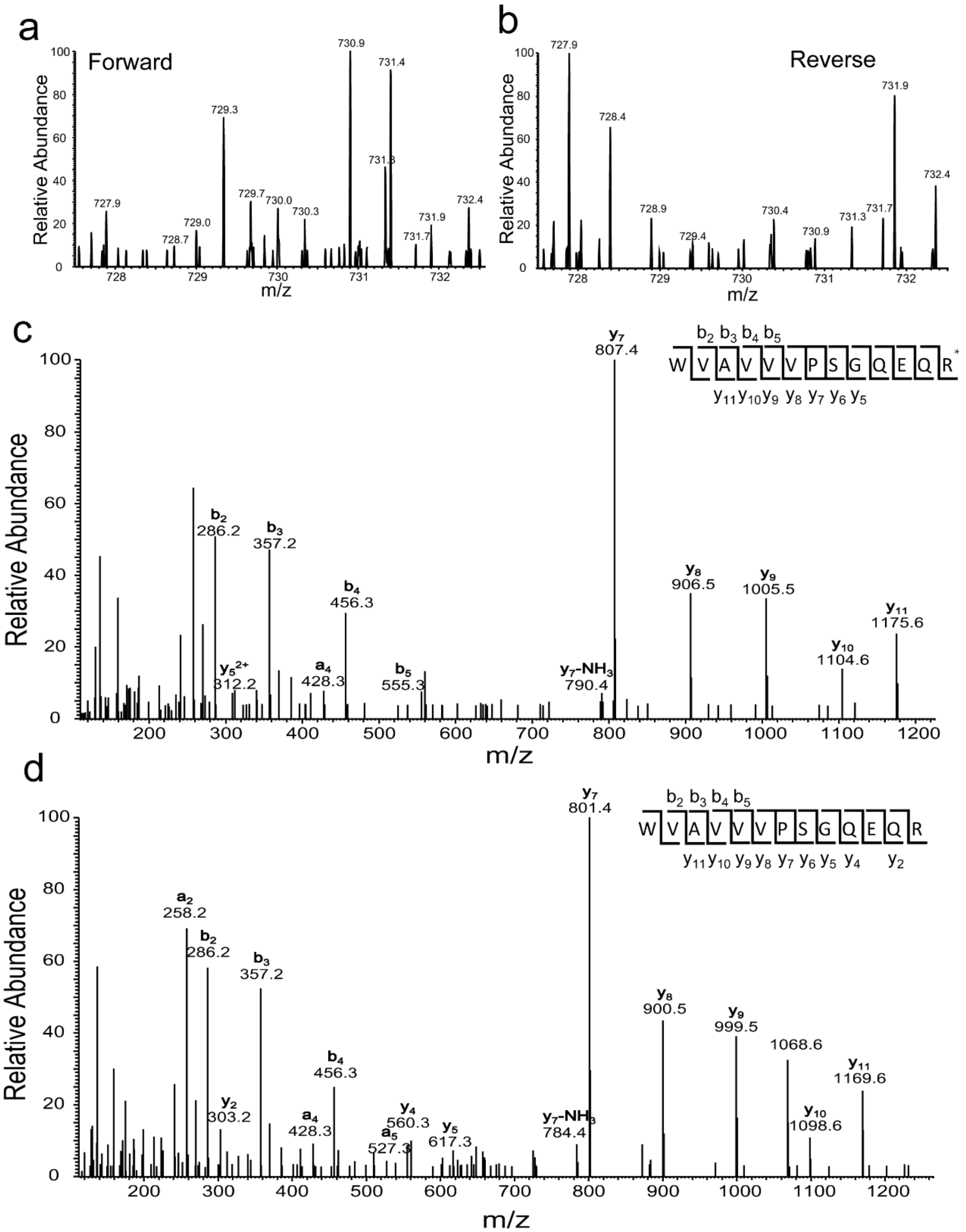

Figure S8. Representative ESI-MS and MS/MS of a tryptic peptide from HLA-A . R* designates the heavy arginine. Shown in (a) and (b) are the ESI-MS for the heavy (m/z 730.9 for the monoisotopic peak of the [M+2H]$^{2+}$ ion) and the light (m/z 727.9 for the monoisotopic peak of the [M+2H]$^{2+}$ ion) arginine -containing peptide observed in forward and reverse SILAC experiments. Displayed in (c) and (d) are the MS/MS for the [M+2H]$^{2+}$ ion s of the heavy- and light- arginine -bearing peptide.

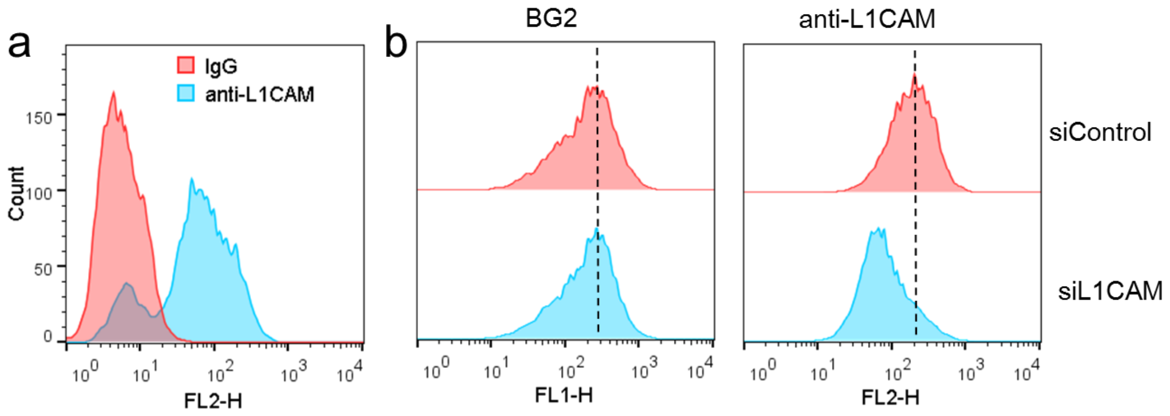

Figure S9. The relationship between the BG2 and L1CAM. (a) Flow cytometry assay results showed the expression of L1CAM on SH-SY5Y cells. IgG, which do not bind to cells, was used as negative control. (b) Flow cytometry assay of siRNA-mediated L1CAM knockdown experiment. Binding toward BG2 and anti-L1CAM were assessed at 72 h after siRNA treatment, where reduced binding toward anti-L1CAM indicated the successful knock-down of L1CAM after siRNA treatment.

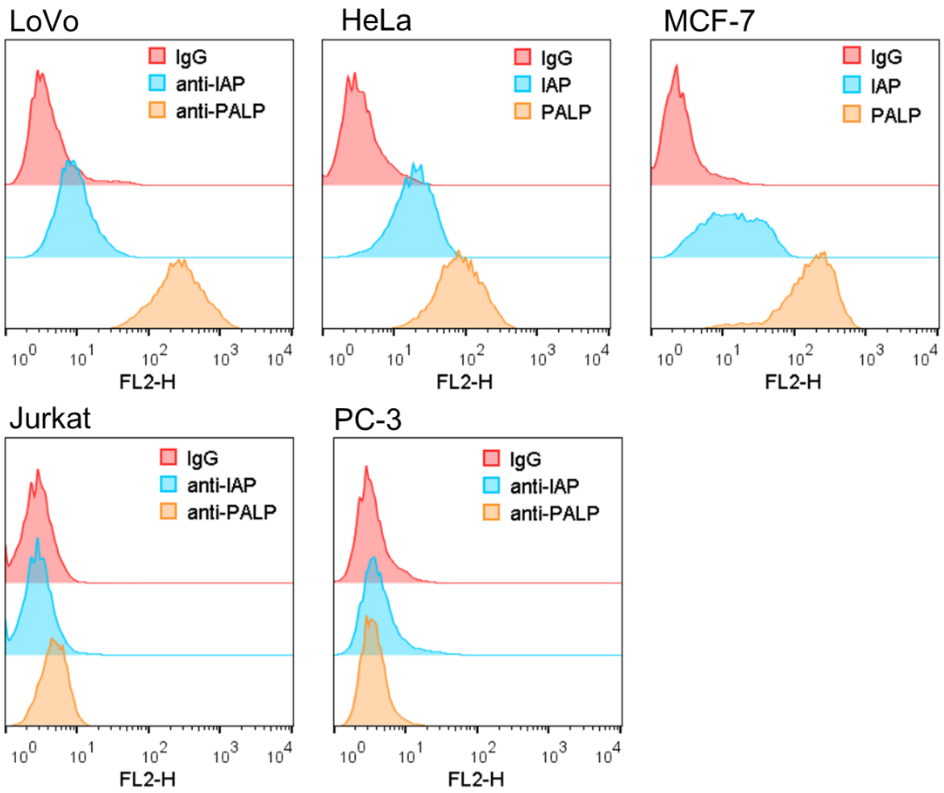

Figure S10. The expression of IAP and PLAP on LoVo, Hela, MCF-7, Jurkat and PC3 cell lines. IgG does not bind to cells, was used as negative control.

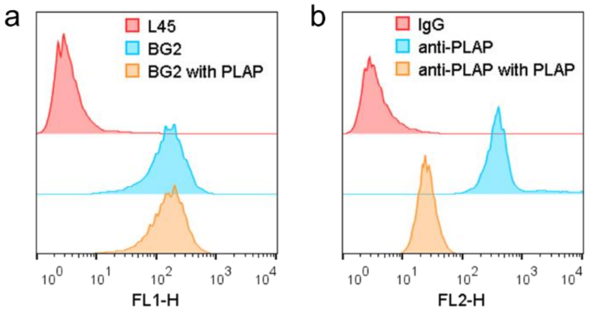

Figure S11. The competition experiment using purified human PLAP. (a) The binding assay of BG2 to LoVo cells in the absence and presence of PLAP protein. (b) The binding assay of anti-PLAP to LoVo cells in the absence and presence of PLAP protein. L45 was the control sequence.

Table S1. The DNA sequences used in the paper.

Name	sequences(5'to 3')
BG2	CAAGGAATAGGGGTCGGTGTGGGTGGTTATGATTGGCTTCCTTG
BG2m	CAAGGAATAGGGGTCGGTGTGGGTGGTGGCTTCCTTG
BG2c	TAGGGGTCGGTGTGGGTGGTTATGATTGGC
BG2c2	TAGGGGTCGGTGTGGGTGGTTATGATT
BG2c3	TCGGTGTGGGTGGTTATGATTGGC
BG2t	CAAGGAATAttttTCttTGTtttTttTTATGATTttCTTCCTTG
BG2a	TCAAGGAATAGGGGTCaGTGTGGGTGGTTATGATTGGCTTCCTTG
BG4	TAAAAAATAGGGGTCGGTGTGGGTGGTTATGATTGGCTTTTTTA
BG2-AF647	AF647-TCAAGGAATAGGGGTCGGTGTGGGTGGTTATGATTGGCTTCCTTGT* T*
L45-AF647	AF647-NNNNNNNNNNNNNNNNNNNNNNNNNNNNNNNNNNNNNNNNNNNNN T* T*
BC1	TGTCCGTGTTTTTGGTTTGCTGCGGTGGGCTCATGGAC

* Phosphorthioated bases

Table S2. The candidate proteins of aptamer BG2, endogenously biotinylated proteins and other proteins identified using SILAC -based quantitative proteomic analysis.

Protein ID	Protein names	Gene names	Unique peptides	Sequence coverage [%]	Score	Ratio (BG2/BC1)
Specific binding proteins toward aptamer BG2						
P09923	Intestinal-type alkaline phosphatase	ALPI	22	60	323.31	>20
P05187	placental type alkaline phosphatase	ALPP	13	68.6	323.31	>20
P10696	Germ cell alkaline phosphatase	ALPPL2	2	51.3	24.046	>20
P32004	Neural cell adhesion molecule L1	L1CAM	6	5.5	53.975	>20
P01891	HLA class I histocompatibility antigen, A-68 alpha chain	HLA-A	1	8.3	24.706	>20
Endogenously biotinylated proteins						

Protein ID	Protein names	Gene names	Unique peptides	Sequence coverage [%]	Score	Ratio (BG2/BC1)
P05166	Propionyl-CoA carboxylase beta chain, mitochondrial	PCCB	31	73.7	323.31	1.06±0.04
P05165	Propionyl-CoA carboxylase alpha chain, mitochondrial	PCCA	40	63	323.31	1.06±0.05
Q96RQ3	Methylcrotonoyl-CoA carboxylase subunit alpha, mitochondrial	MCCC1	35	65.9	323.31	1.02±0.02
P11498	Pyruvate carboxylase	PC	70	72.3	323.31	1.00±0.02
Q9HCC0	Methylcrotonoyl-CoA carboxylase beta chain, mitochondrial	MCCC2	30	61.6	323.31	0.94±0.02
B2ZZ90	Acetyl-CoA carboxylase 1	ACACA	41	56.3	323.31	0.94±0.05
Other proteins						
Q99714	3-hydroxyacyl-CoA dehydrogenase type-2	HSD17B10	5	33	73.823	1.16±0.18
P24752	Acetyl-CoA acetyltransferase, mitochondrial	ACAT1	6	21.5	69.343	1.02±0.08
P30084	Enoyl-CoA hydratase, mitochondrial	ECHS1	1	5.9	9.9468	1.01±0.50
Q6IAV5	Succinyl-CoA:3-ketoacid-coenzyme A transferase	OXCT	4	10.2	25.782	0.97±0.06
P12956	X-ray repair cross-complementing protein 6	XRCC6	41	55.7	323.31	21.81±15.22
P13010	X-ray repair cross-complementing protein 5	XRCC5	44	65.2	323.31	20.72±13.82
Q96BA7	Heterogeneous nuclear ribonucleoprotein U	HNRNPU	11	16.6	109.66	3.40±0.35
D6R9P3	Heterogeneous nuclear ribonucleoprotein A/B	HNRNPAB	3	15	66.631	2.35±0.49
H0YA96	Heterogeneous nuclear ribonucleoprotein D0	HNRNPD	3	18.6	19.809	1.91±0.07
F5GZS6	4F2 cell-surface antigen heavy chain	SLC3A2	6	10.7	54.99	1.47±0.27
F8WE65	Peptidyl-prolyl cis-trans isomerase	PPIA	2	15	11.537	1.47±0.08
Q53FB6	Aldehyde dehydrogenase, mitochondrial	ALDH2	6	12.8	41.481	1.30±0.01
P18085	ADP-ribosylation factor 4	ARF4	2	38.3	45.095	1.23±0.12
Q06830	Peroxiredoxin-1	PRDX1	5	27.5	41.466	1.21±0.09
P04792	Heat shock protein beta-1	HSPB1	4	22	57.013	1.17±0.30
P23526	Adenosylhomocysteinase	AHCY	3	9	20.346	1.16±0.37
Q67FW5	UDP-GlcNAc:betaGal beta-1,3-N-acetylglucosaminyltransferase-like protein 1	B3GNTL1	1	4.7	6.9098	1.13±0.01
Q9UPE4	Mitochondrial import inner membrane translocase subunit TIM44	hTIM44	5	13.5	32.38	1.12±0.23
P60842	Eukaryotic initiation factor 4A-I	EIF4A1	3	8.6	25.104	1.12±0.27
P14174	Macrophage migration inhibitory factor	MIF	2	17.4	62.716	1.12±0.19
Q16380	Metastasis-associated gene protein (Fragment)	metastasis-associated gene	1	53.8	16.761	1.10±0.19
P22695	Cytochrome b-c1 complex subunit 2	UQCRC2	3	12.4	32.478	1.10±0.13
P36776	Lon protease homolog, mitochondrial	LONP1	8	14	56.771	1.08±0.58
B3KTN4	Citrate synthase;Citrate synthase	CS	4	10	33.453	1.08±0.36
Q08ES8	60S ribosomal protein L11	RPL11	2	13	34.791	1.08±0.21
P63244	Guanine nucleotide-binding protein subunit beta-2-like 1	GNB2L1	13	49.5	203.9	1.07±0.25
P07355	Annexin A2	ANXA2	5	40.9	48.006	1.07±0.045
P61604	10 kDa heat shock protein, mitochondrial	HSPE1	5	52.6	41.316	1.06±0.34
Q9BTQ7	60S ribosomal protein L23	RPL23	5	45.5	71.45	1.06±0.20
P05141	ADP/ATP translocase 2	SLC25A5	7	21.5	52.42	1.06±0.29
P11142	Heat shock cognate 71 kDa protein	HSPA8	3	18.3	44.179	1.05±0.16
P49448	Glutamate dehydrogenase	GLUD1	6	15.9	47.849	1.05±0.14
P42765	3-ketoacyl-CoA thiolase, mitochondrial	ACAA2	8	25.6	155.18	1.05±0.14
B4DMA2	Heat shock protein HSP 90-beta	HSP90AB1	12	27.4	181.93	1.05±0.19
P00505	Aspartate aminotransferase	GOT2	6	22.4	146.48	1.04±0.25
P57721	Poly(rC)-binding protein 3	PCBP3	2	25.6	50.722	1.04±0.22
P62263	40S ribosomal protein S14	RPS14	2	15.9	18.821	1.04±0.45
I3L3P7	40S ribosomal protein S15a	RPS15A	4	40	30.154	1.03±0.50
P40926	Malate dehydrogenase	MDH2	9	40	105.72	1.03±0.20

Protein ID	Protein names	Gene names	Unique peptides	Sequence coverage [%]	Score	Ratio (BG2/BC1)
P30048	Thioredoxin-dependent peroxide reductase, mitochondrial	PRDX3	4	24.2	45.504	1.03±0.10
A2VCT2	2-oxoglutarate dehydrogenase, mitochondrial	OGDH	4	5	29.342	1.02±0.19
P62826	GTP-binding nuclear protein Ran	RAN	8	36.9	122.61	1.02±0.17
P04406	Glyceraldehyde-3-phosphate dehydrogenase	GAPDH	8	34.6	152.45	1.02±0.17
A8K5I0	Heat shock 70 kDa protein 1B	HSPA1B	2	15.9	80.503	1.09±0.21
S4R3Z2	Aldo-keto reductase family 1 member C3	AKR1C3	3	11.7	18.815	1.01±0.26
Q96RE1	Elongation factor 1-alpha	EEF1A1L14	6	15.1	91.047	1.01±0.22
H0YML5	Phosphoenolpyruvate carboxykinase [GTP], mitochondrial	PCK2	9	20	64.562	1.01±0.11
Q8WYJ5	Histidine triad nucleotide-binding protein 2, mitochondrial	HINT2	4	53.1	25.478	1.01±0.09
P10620	Microsomal glutathione S-transferase 1	MGST1	1	15.1	12.95	1.00±0.15
Q96KX2	F-actin-capping protein subunit alpha-3	CAPZA3	1	2.7	6.7945	1.00±0.06
P06733	Alpha-enolase;Enolase	ENO1	7	22.1	89.076	1.00±0.08
P25705	ATP synthase subunit alpha	ATP5A1	16	36.3	288.76	1.00±0.06
Q9NSE4	Isoleucine--tRNA ligase, mitochondrial	IARS2	10	14.8	66.117	1.00±0.10
B4DGK4	Alternative protein SYT7	SYT7	1	6.6	6.6097	1.00±0.04
E7D7Y0	Pyrroline-5-carboxylate reductase	PYCR1	2	11.3	68.071	0.99±0.16
M0R1V7	Ubiquitin-60S ribosomal protein L40	UBA52	2	39.7	28.104	0.97±0.27
Q53SS8	Poly(rC)-binding protein 1	PCBP1	1	7	12.381	0.97±0.23
B4DJ81	NADH-ubiquinone oxidoreductase 75 kDa subunit, mitochondrial	NDUFS1	2	4.9	12.629	0.97±0.17
P49411	Elongation factor Tu, mitochondrial	TUFM	24	56.4	323.31	0.97±0.02
Q99798	Aconitate hydratase, mitochondrial	ACO2	4	11.8	35.503	0.96±0.25
P31040	Succinate dehydrogenase [ubiquinone] flavoprotein subunit, mitochondrial	SDHA	10	21.7	166.75	0.95±0.23
B4DJ63	Serine hydroxymethyltransferase;Serine hydroxymethyltransferase, mitochondrial	SHMT2	7	18.8	65.903	0.95±0.07
P10809	60 kDa heat shock protein, mitochondrial	HSPD1	29	55.3	323.31	0.95±0.15
P43897	Elongation factor Ts, mitochondrial;Elongation factor Ts	TSFM	2	10.5	39.179	0.94±0.25
P63261	Actin, cytoplasmic 2	ACTG1	10	29.4	138.6	0.94±0.53
P07437	Tubulin beta chain	TUBB	1	26.5	115.32	0.93±0.28
P49327	Fatty acid synthase	FASN	8	4.3	85.811	0.93±0.37
H3BT58	Coactosin-like protein	COTL1	2	32.9	21.076	0.93±0.14
Q16698	2,4-dienoyl-CoA reductase, mitochondrial	DECR1;DECR	4	17.3	40.4	0.93±0.28
P42677	40S ribosomal protein S27	RPS27	3	38.1	64.561	0.93±0.18
Q14914	Prostaglandin reductase 1	LTB4DH; PTGR1	3	10.6	25.174	0.93±0.08
Q01991	Acetyltransferase component of pyruvate dehydrogenase complex	DLAT	1	6.8	18.611	0.92±0.06
Q9BV61	TRAP1 protein (Fragment)	TRAP1	1	22.9	263.17	0.92±0.16
P54886	Delta-1-pyrroline-5-carboxylate synthase	ALDH18A1	20	29.2	240.42	0.92±0.08
P31327	Carbamoyl-phosphate synthase [ammonia], mitochondrial	CPS1	78	59.3	323.31	0.92±0.11
P14618	Pyruvate kinase PKM	PKM	13	31.6	143.07	0.91±0.23
P22626	Heterogeneous nuclear ribonucleoproteins A2/B1	HNRNPA2B1	2	7.3	15.225	0.91±0.41
P68363	Tubulin alpha-1B chain	TUBA1B	11	36.1	194.97	0.91±0.18
P23396	40S ribosomal protein S3	RPS3	5	23	42.692	0.90±0.35
P42704	Leucine-rich PPR motif-containing protein, mitochondrial	LRPPRC	28	22	296.87	0.89±0.15
Q96EY1	DnaJ homolog subfamily A member 3, mitochondrial	DNAJA3	5	12.1	89.394	0.88±0.09
H0YM48	ATP-dependent Clp protease ATP-binding subunit clpX-like, mitochondrial	CLPX	5	8.5	40.477	0.88±0.09

Protein ID	Protein names	Gene names	Unique peptides	Sequence coverage [%]	Score	Ratio (BG2/BC1)
H0YJ63	Activator of 90 kDa heat shock protein ATPase homolog 1	AHSA1	1	24.4	27.545	0.88 ± 0.14
Q0QEN7	ATP synthase subunit beta;ATP synthase subunit beta, mitochondrial	ATP5B	8	22	80.931	0.87 ± 0.05
Q53TX0	Glutaminase kidney isoform, mitochondrial	GLS	4	15.3	38.13	0.87 ± 0.14
P60866	40S ribosomal protein S20	RPS20	2	19.3	11.728	0.86 ± 0.20
P50213	Isocitrate dehydrogenase [NAD] subunit, mitochondrial	IDH3A	1	11	13.05	0.85 ± 0.22
P84095	Rho-related GTP-binding protein RhoG	RHOG	4	25.7	51.373	0.83 ± 0.13
A3KPC7	Histone H2A	HIST1H2AH	4	32.8	40.017	0.83 ± 0.53
L0R512	Alternative protein NCAM2	NCAM2	1	21.4	7.6101	0.83 ± 0.51
P36957	Dihydrolipoyllysine-residue succinyltransferase component of 2-oxoglutarate dehydrogenase complex, mitochondrial	DLST	3	7.3	23.44	0.83 ± 0.11
P33778	Histone H2B	HIST1H2BB	6	46.8	87.022	0.83 ± 0.50
P61026	Ras-related protein Rab-10	RAB10	2	11	18.131	0.82 ± 0.22
P62805	Histone H4	HIST1H4H	8	59.2	167.4	0.80 ± 0.49
P08670	Vimentin	VIM	8	27.1	125.67	0.79 ± 0.51
P38646	Stress-70 protein, mitochondrial	HSPA9	26	40.9	323.31	0.79 ± 0.21
B2RD24	Monofunctional C1-tetrahydrofolate synthase, mitochondrial	MTHFD1L	7	8.9	45.877	0.76 ± 0.29
P21333	Filamin-A	FLNA	5	3.2	31.23	0.72 ± 0.21
P68431	Histone H3.1	HIST1H3A	1	40.4	27.745	0.63 ± 0.55
G3V576	Heterogeneous nuclear ribonucleoproteins C1/C2	HNRNPC	6	26	49.548	0.59 ± 0.28
Q567R6	Single-stranded DNA-binding protein	SSBP1	9	58.8	313.62	0.45 ± 0.10
P10412	Histone H1.4	HIST1H1E	1	5.3	9.5776	0.38 ± 0.04
Q13332	Receptor-type tyrosine-protein phosphatase S	PTPRS	10	8.5	136.15	0.13 ± 0.11

References

[1] N. de-los-Santos-Álvarez, A. J. Miranda-Ordieres, P. Tuñón-Blanco, *TrAC-Trend. Anal. Chem.* **2008**, *27*, 437.

[2] T. Bing, W. Zheng, X. Zhang, L. Shen, X. Liu, F. Wang, J. Cui, Z. Cao, D. Shangguan, *Sci. Rep.* **2017**, *7*, 15467.

[3] Y. Wang, Y. Luo, T. Bing, Z. Chen, M. Lu, N. Zhang, D. Shangguan, X. Gao, *Plos One* **2014**, *9*, e100243; T. Bing, T. Chang, X. Yang, H. Mei, X. Liu, D. Shangguan, *Bioorg. Med. Chem.* **2011**, *19*, 4211; W.-M. Li, T. Bing, J.-Y. Wei, Z.-Z. Chen, D.-H. Shangguan, J. Fang, *Biomaterials* **2014**, *35*, 6998; C. Qi, T. Bing, H. Mei, X. Yang, X. Liu, D. Shangguan, *Biosens. Bioelectron.* **2013**, *41*, 157; H. Mei, T. Bing, X. Yang, C. Qi, T. Chang, X. Liu, Z. Cao, D. Shangguan, *Anal. Chem.* **2012**, *84*, 7323.

[4] T. Bing, X. Yang, H. Mei, Z. Cao, D. Shangguan, *Bioorg. Med. Chem.* **2010**, *18*, 1798.

[5] M. Zuker, *Nucleic Acids Res.* **2003**, *31*, 3406.

[6] N. Zhang, T. Bing, L. Y. Shen, R. S. Song, L. L. Wang, X. J. Liu, M. R. Liu, J. Li, W. H. Tan, D. H. Shangguan, *Angew. Chem. Int. Ed.* **2016**, *55*, 3914.

[7] J. H. Davis, J. W. Szostak, *Proc. Natl. Acad. Sci. USA* **2002**, *99*, 11616.

[8] K. M. Ruff, T. M. Snyder, D. R. Liu, *J. Am. Chem. Soc.* **2010**, *132*, 9453.
[9] D. Shangguan, T. Bing, N. Zhang, *in Aptamers Selected by Cell-SELEX for Theranostics, Springer Berlin Heidelberg*, **2015**, 13.
[10] Y. R. Wu, K. Sefah, H. P. Liu, R. W. Wang, W. H. Tan, *Proc. Natl. Acad. Sci. USA* **2010**, *107*, 5; K. Sefah, Z. W. Tang, D. H. Shangguan, H. Chen, D. Lopez-Colon, Y. Li, P. Parekh, J. Martin, L. Meng, J. A. Phillips, Y. M. Kim, W. H. Tan, *Leukemia* **2009**, *23*, 235.
[11] J. Bordeaux, A. W. Welsh, S. Agarwal, E. Killiam, M. T. Baquero, J. A. Hanna, V. K. Anagnostou, D. L. Rimm, *Biotechniques* **2010**, *48*, 197.
